# Supplementary material for: Using Mediation Analysis to Understand How Treatments for Paediatric Pain Work: A Systematic Review and Recommendations for Future Research
Source: Children (Basel). 2021 Feb 16;8(2):147. doi: 10.3390/children8020147 (PMC7919827; doi:10.3390/children8020147)
Supplement: Supplementary file 1 [file children-08-00147-s001.pdf]

Supplemental Online Content

Leake, HB, Moseley, GL, Stanton, TR, Heathcote, LC, Pate, JW, Wewege, MA, Lee, H.

Using mediation analysis to understand how treatments for paediatric pain work: A systematic review and recommendations for future research

Supplementary Table S1. Deviations from pre-registered protocol.

Supplementary Table S2. Complete search strategies for electronic databases.

Supplementary Table S3. Reasons for exclusion of full text articles.

Supplementary Table S4. Summary of mediation analyses using parent-reported outcomes.

Supplementary Table S5. Results of mediation analyses using parent-reported outcomes.

Supplementary Table S6. Justifications of study quality assessment.

Supplementary Table S7. Findings of mediation analyses of included studies.

eReferences

This supplementary material has been provided by the authors to give readers additional information about their work.

Supplementary Table S1 Deviations from pre-registered protocol

Date	Section	Original protocol	Revised protocol	Rationale
11/12/2019	Quality assessment	Reviewers will appraise methodological quality of studies using a critical appraisal tool for mediation studies developed by Mansell et al. (2013).	Reviewers will appraise methodological quality of studies using a framework of recommendations adapted from Vo et al. (2020).	Study published since date protocol was registered, provides a quality assessment tool that is more relevant that the original.
22/04/2020	Grouping of studies for narrative synthesis	No description of grouping studies in data synthesis	We will group studies based on duration of pain, either as acute pain (less than 3 months duration) or chronic pain (three months or long duration) (Merskey et al., 1994).	This decision reflects the assumption that the mechanisms of treatment effect may depend on pain duration.

Supplementary Table S2. Complete search strategies for electronic databases.

MEDLINE (OvidSP)

1	mediat*.mp.
2	structural equation model?ing.mp.
3	(Baron and Kenny).mp.
4	product of coefficient.mp.
5	difference in coefficient.mp.
6	process of change.mp.
7	causal pathway.mp.
8	indirect effect.mp.
9	process variable.mp.
10	(process adj2 evaluation).mp.
11	1 or 2 or 3 or 4 or 5 or 6 or 7 or 8 or 9 or 10
12	exp pain/
13	fibromyalgia/
14	juvenile idiopathic arthritis/
15	exp Complex Regional Pain Syndromes/
16	migraine disorders/
17	exp headache disorders/
18	Irritable Bowel Syndrome/
19	(pain* or fibromyalgia* or crps or head?ache* or migraine* or cephalgi* or stomach?ache* or tummy?ache* or abdominal?ache* or belly?ache* or "irritable bowel syndrome" or arthralgia or (juvenile adj2 arthrit*)).mp.
20	12 or 13 or 14 or 15 or 16 or 17 or 18 or 19
21	exp Child/
22	Adolescent/
23	Pediatrics/
24	(Child* or adolescen* or juvenil* or teen* or p?ediatric* or youth* or "young person*" or "young adult*").mp.
25	21 or 22 or 23 or 24
26	11 and 20 and 25
27	limit 26 to humans
28	limit 26 to animals
29	26 not 28
30	27 or 29

EMBASE (OvidSP)

1	mediat*.mp.
2	"structural equation model?ing".mp.
3	(Baron and Kenny).mp.
4	product of coefficient.mp.
5	difference in coefficient.mp.
6	process of change.mp.
7	causal pathway.mp.
8	indirect effect.mp.
9	process variable.mp.
10	(process adj2 evaluation).mp.
11	1 or 2 or 3 or 4 or 5 or 6 or 7 or 8 or 9 or 10
12	exp pain/
13	fibromyalgia/
14	exp juvenile idiopathic arthritis/
15	exp complex regional pain syndrome/
16	exp migraine/
17	Headache/
18	irritable colon/
19	(pain* or fibromyalgia* or crps or head?ache* or migraine* or cephalgi* or stomach?ache* or tummy?ache* or abdominal?ache* or belly?ache* or "irritable bowel syndrome" or arthralgia or (juvenile adj2 arthrit*)).mp.
20	12 or 13 or 14 or 15 or 16 or 17 or 18 or 19
21	exp child/
22	exp adolescent/
23	pediatrics/
24	(Child* or adolescen* or juvenil* or teen* or p?ediatric* or youth* or "young person*" or "young adult*").mp.
25	21 or 22 or 23 or 24
26	11 and 20 and 25
27	limit 26 to humans
28	limit 26 to animals
29	26 not 28
30	27 or 29

PsycINFO (OvidSP)

1	mediat*.mp.
2	structural equation model?ing.mp.
3	(Baron and Kenny).mp.
4	product of coefficient.mp.
5	difference in coefficient.mp.
6	process of change.mp.
7	causal pathway.mp.
8	indirect effect.mp.
9	process variable.mp.
10	(process adj2 evaluation).mp.
11	1 or 2 or 3 or 4 or 5 or 6 or 7 or 8 or 9 or 10
12	exp pain/
13	fibromyalgia/
14	exp arthritis/
15	exp "complex regional pain syndrome (type i)"/
16	exp headache/
17	irritable bowel syndrome/
18	(pain* or fibromyalgia* or crps or head?ache* or migraine* or cephalgi* or stomach?ache* or tummy?ache* or abdominal?ache* or belly?ache* or "irritable bowel syndrome" or arthralgia or (juvenile adj2 arthrit*)).mp.
19	12 or 13 or 14 or 15 or 16 or 17 or 18
20	pediatrics/
21	(Child* or adolescen* or juvenil* or teen* or p?ediatric* or youth* or "young person*" or "young adult*").mp.
22	20 or 21
23	11 and 19 and 22
24	limit 23 to human
25	limit 23 to animal
26	23 not 25
27	24 or 26

Emcare (OvidSP)

1	mediat*.mp.
2	structural equation model?ing.mp.
3	(Baron and Kenny).mp.
4	product of coefficient.mp.
5	difference in coefficient.mp.
6	process of change.mp.
7	causal pathway.mp.
8	indirect effect.mp.
9	process variable.mp.
10	(process adj2 evaluation).mp.
11	1 or 2 or 3 or 4 or 5 or 6 or 7 or 8 or 9 or 10
12	exp pain/
13	fibromyalgia/
14	exp juvenile rheumatoid arthritis/
15	exp complex regional pain syndrome/
16	irritable colon/
17	(pain* or fibromyalgia* or crps or head?ache* or migraine* or cephalgi* or stomach?ache* or tummy?ache* or abdominal?ache* or belly?ache* or "irritable bowel syndrome" or arthralgia or (juvenile adj2 arthrit*)).mp.
18	12 or 13 or 14 or 15 or 16 or 17
19	exp child/
20	exp adolescent/
21	pediatrics/
22	(Child* or adolescen* or juvenil* or teen* or p?ediatric* or youth* or "young person*" or "young adult*").mp.
23	19 or 20 or 21 or 22
24	11 and 18 and 23
25	limit 24 to human
26	limit 24 to animal
27	24 not 26
28	25 or 27

Cochrane Central Register of Controlled Trials (CENTRAL)

1	mediat*
2	structural equation modelling
3	structural equation modeling
4	Baron and Kenny
5	product of coefficient
6	difference in coefficient
7	process of change
8	casual pathway
9	indirect effect
10	process variable
11	process NEAR/2 evaluation
12	#1 OR #2 OR #3 OR #4 OR #5 OR #6 OR #7 #9 OR #10 OR #11
13	MeSH descriptor: [Pain] explode all trees
14	MeSH descriptor: [Fibromyalgia] explode all trees
15	MeSH descriptor: [Arthritis, Juvenile] explode all trees
16	MeSH descriptor: [Complex Regional Pain Syndromes] explode all trees
17	MeSH descriptor: [Headache Disorders] explode all trees
18	MeSH descriptor: [Irritable Bowel Syndrome] explode all trees
19	(pain* or fibromyalgia* or crps or head?ache* or migraine* or cephalgi* or stomach*ache* or tummy*ache* or abdominal*ache* or belly*ache* or "irritable bowel syndrome" or arthralgia or (juvenile NEAR/2 arthrit*))
20	#13 OR #14 OR #15 OR #16 OR #17 OR #19
21	MeSH descriptor: [Child] explode all trees
22	MeSH descriptor: [Adolescent] explode all trees
23	MeSH descriptor: [Pediatrics] explode all trees
24	(Child* or adolescen* or juvenil* or teen* or p*ediatric* or youth* or "young person" or "young adult")
25	#21 OR #22 OR #24
26	#12 AND #20 AND #25
	Limit to *Trials*

Supplementary Table S3. Reasons for exclusion of full text articles [*ordered alphabetically by first author*]. Full references are provided in eReferences.

**Study**	**Reason for exclusion**
Absoud et al. (2017)	Study population did not have pain at baseline
Allantaz et al. (2007)	Study design was irrelevant to this review
Allen et al. (1998)	No statistical test for mediation performed
Allison et al. (2016)	Participants not paediatric (3-18 years)
Altieri et al. (2020)	No statistical test for mediation performed
Anand et al. (2011)	Participants not paediatric (3-18 years)
Angst et al. (2012)	Participants not paediatric (3-18 years)
Atlas et al. (2014)	Participants not paediatric (3-18 years)
Baccini et al. (2017)	Participants not paediatric (3-18 years)
Baildam et al. (2013)	No statistical test for mediation performed
Barber et al. (1977)	Participants not paediatric (3-18 years)
Barlow et al. (2014)	Study design was irrelevant to this review
Beal et al. (2020)	Does not investigate the effects of an intervention
Bentley et al. (2005)	Participants not paediatric (3-18 years)
Blackwell et al. (2012)	Study design was irrelevant to this review
Bonnert et al. (2017)	Study design was irrelevant to this review
Bowers et al. (2016)	Study design was irrelevant to this review
Brennan et al. (1991)	Participants not paediatric (3-18 years)
Brown et al. (2019)	Study design was irrelevant to this review
Brown et al. (2012)	No statistical test for mediation performed
Brunner et al. (2012)	No statistical test for mediation performed
Bryskin et al. (2015)	No statistical test for mediation performed
Buenaver et al. (2012)	Study design was irrelevant to this review
Caixeta et al. (2020)	No statistical test for mediation performed
Cakar Turhan et al. (2015)	No statistical test for mediation performed
Cappucci et al. (2015)	Study design was irrelevant to this review
Castien et al. (2013)	Participants not paediatric (3-18 years)
Chan et al. (2017)	Study design was irrelevant to this review

Chou et al. (2011)	Participants not paediatric (3-18 years)
Christidis et al. (2015)	Participants not paediatric (3-18 years)
Clementi et al. (2020)	Does not compare two or more groups
Conti et al. (2020)	Participants not paediatric (3-18 years)
Corinaldesi et al. (2009)	Participants not paediatric (3-18 years)
Cunningham et al. (2020)	No statistical test for mediation performed
Dekker et al. (2016)	Study design was irrelevant to this review
DiVasta et al. (2015)	No statistical test for mediation performed
Du et al. (2018)	No statistical test for mediation performed
Dura-Ferrandis et al. (2017)	Participants not paediatric (3-18 years)
Essner (2013)	Study design was irrelevant to this review
Evans et al. (2014)	Study design was irrelevant to this review
Evans et al. (2011)	No statistical test for mediation performed
Evans et al. (2017)	Study design was irrelevant to this review
Evans et al. (2016)	Study design was irrelevant to this review
Evans et al. (2006)	Study design was irrelevant to this review
Fales et al. (2020)	No intervention
Ferrari et al. (2010)	Participants not paediatric (3-18 years)
Field et al. (2020)	No statistical test for mediation performed
Finch et al. (2009)	Participants not paediatric (3-18 years)
Fiorelli et al. (2010)	Participants not paediatric (3-18 years)
Fisher et al. (2016)	Does not compare two or more groups
Forsythe et al. (2011)	Study design was irrelevant to this review
Foxen-Craft (2017)	Study design was irrelevant to this review
Garland et al. (2012)	Participants not paediatric (3-18 years)
Gaultney (2020)	Study design was irrelevant to this review
Ghorbani et al. (2020)	Participants not paediatric (3-18 years)
Gillis (2002)	Study design was irrelevant to this review
Glenn et al. (2014)	Study design was irrelevant to this review
Gomez-Mancilla et al. (2001)	Participants not paediatric (3-18 years)

Gottschlich et al. (2011)	No statistical test for mediation performed
Grinsvall et al. (2015)	Study design was irrelevant to this review
Vos et al. (2017)	Participants not paediatric (3-18 years)
Harel et al. (2004)	No statistical test for mediation performed
Harper et al. (2012)	Study design was irrelevant to this review
Hashish et al. (1988)	Participants not paediatric (3-18 years)
He et al. (2015)	Study design was irrelevant to this review
Hechler et al. (2010)	No statistical test for mediation performed
Heeney et al. (2016a)	No statistical test for mediation performed
Heeney et al. (2018)	Study design was irrelevant to this review
Heeney et al. (2016b)	No statistical test for mediation performed
Herroeder et al. (2007)	Participants not paediatric (3-18 years)
Hildenbrand et al. (2020)	Independent variable in the mediation analysis was not the intervention
Hillgrove-Stuart et al. (2013)	Participants not paediatric (3-18 years)
Hilt (2009)	Study design was irrelevant to this review
Hind et al. (2017)	No statistical test for mediation performed
Hooke et al. (2018)	Does not compare two or more groups
Hoyeraal et al. (1978)	No statistical test for mediation performed
Ingelmo et al. (2007)	No statistical test for mediation performed
Janssens et al. (2014)	Does not compare two or more groups
Jones et al. (2018)	Study design was irrelevant to this review
Jonsbu et al. (2011)	Participants not paediatric (3-18 years)
Junghans-Rutelonis et al. (2018)	Independent variable in the mediation analysis was not the intervention
Khayat et al. (2015)	Participants not paediatric (3-18 years)
Kilkens et al. (2005)	Participants not paediatric (3-18 years)
Kobayashi et al. (2017)	Study design was irrelevant to this review
Kościelniak-Merak et al. (2020)	No statistical test for mediation performed
Lai et al. (2019)	No statistical test for mediation performed

Langer et al. (2014)	Study design was irrelevant to this review
Langer et al. (2013)	Study design was irrelevant to this review
Lee et al. (2020)	No statistical test for mediation performed
Leeuw et al. (2008)	Participants not paediatric (3-18 years)
Levy et al. (2012)	Study design was irrelevant to this review
Lewis et al. (1996)	Study design was irrelevant to this review
Li et al. (2016)	Participants not paediatric (3-18 years)
Lim et al. (2019)	Study design was irrelevant to this review
Liossi et al. (2007)	Does not compare two or more groups
Lohsiriwat et al. (2004)	Participants not paediatric (3-18 years)
Love et al. (2019)	Study design was irrelevant to this review
Lowen et al. (2013)	Participants not paediatric (3-18 years)
Lu et al. (2013)	Study design was irrelevant to this review
Luciano et al. (2014)	Participants not paediatric (3-18 years)
Lustig et al. (1996)	Study design was irrelevant to this review
Maddison et al. (2006)	Participants not paediatric (3-18 years)
Malattia et al. (2020)	No statistical test for mediation performed
McGarrigle et al. (2018)	Study design was irrelevant to this review
Meier et al. (2009)	No statistical test for mediation performed
Melzack et al. (1980a)	Participants not paediatric (3-18 years)
Melzack et al. (1980b)	Participants not paediatric (3-18 years)
Miller et al. (2017)	Study design was irrelevant to this review
Milling et al. (2006)	Participants not paediatric (3-18 years)
Milling et al. (2007)	Participants not paediatric (3-18 years)
Mohammed et al. (2010)	Study design was irrelevant to this review
Moore et al. (1992)	Participants not paediatric (3-18 years)
Mulroy et al. (2011)	No statistical test for mediation performed
Neville et al. (2020)	No intervention
Nickel et al. (2012)	Participants not paediatric (3-18 years)
Niedermann et al. (2011)	Participants not paediatric (3-18 years)

Noel et al. (2015)	Does not compare two or more groups
Noel et al. (2018)	Study design was irrelevant to this review
Palermo et al. (2018)	Study design was irrelevant to this review
Palstam et al. (2016)	Participants not paediatric (3-18 years)
Pavlova et al. (2017)	Study design was irrelevant to this review
Pavlova et al. (2018)	Study design was irrelevant to this review
Pavlova et al. (2020)	Does not investigate the effects of an intervention
Peatfield et al. (1983)	Participants not paediatric (3-18 years)
Petter et al. (2014)	Independent variable in the mediation analysis was not the intervention
Peugh et al. (2017)	Study design was irrelevant to this review
Poppert Cordts et al. (2019)	Study design was irrelevant to this review
Posner (1999)	Study design was irrelevant to this review
Pringsheim et al. (2002)	Participants not paediatric (3-18 years)
Puzino et al. (2018)	Study design was irrelevant to this review
Quispe-Cabanillas et al. (2012)	Participants not paediatric (3-18 years)
Randall et al. (2020)	Does not compare two or more groups
Reddy et al. (2020)	No statistical test for mediation performed
Reed-Knight et al. (2018)	Study design was irrelevant to this review
Reid (2003)	Study design was irrelevant to this review
Reme et al. (2011)	Participants not paediatric (3-18 years)
Riggenbach et al. (2020)	Study design was irrelevant to this review
Robinson et al. (2013)	Study design was irrelevant to this review
Rolli Salathe et al. (2020)	Does not investigate the effects of an intervention
Ruperte et al. (2011)	No statistical test for mediation performed
Ruperto et al. (2013)	No statistical test for mediation performed
Saxe et al. (2006)	Does not compare two or more groups
Schoenen et al. (2013)	Participants not paediatric (3-18 years)
Schreiber et al. (2001)	Participants not paediatric (3-18 years)
Schurman et al. (2012)	Study design was irrelevant to this review

Shah et al. (2002)	No statistical test for mediation performed
Shi et al. (2011)	Participants not paediatric (3-18 years)
Shi et al. (2015)	Participants not paediatric (3-18 years)
Sieberg et al. (2011)	Study design was irrelevant to this review
Simister et al. (2018)	Participants not paediatric (3-18 years)
Slaman et al. (2015)	Participants not paediatric (3-18 years)
Spinhoven et al. (2004)	Participants not paediatric (3-18 years)
Stratelak et al. (1996)	Participants not paediatric (3-18 years)
Taheri et al. (2016)	Study design was irrelevant to this review
Tarnowski et al. (1987)	Study design was irrelevant to this review
ter Kuile et al. (1996)	Participants not paediatric (3-18 years)
Thieme et al. (2015)	Participants not paediatric (3-18 years)
Tran et al. (2015)	Study design was irrelevant to this review
Tran (2015)	Study design was irrelevant to this review
Tremblay et al. (2010)	Study design was irrelevant to this review
Troullos et al. (1990)	Participants not paediatric (3-18 years)
Tsao et al. (2006a)	Study design was irrelevant to this review
Tsao et al. (2006b)	Study design was irrelevant to this review
Turner et al. (1995)	Participants not paediatric (3-18 years)
Tutuncu et al. (2013)	No statistical test for mediation performed
van Tilburg et al. (2017)	Study design was irrelevant to this review
VanDyck et al. (1991)	Participants not paediatric (3-18 years)
Varni et al. (2018)	Study design was irrelevant to this review
Varni et al. (2020)	Study design was irrelevant to this review
Venkatraghavan et al. (2016)	Participants not paediatric (3-18 years)
VerLee et al. (2012)	Study design was irrelevant to this review
Wall (2000)	Study design was irrelevant to this review
Wallace (2009)	Study design was irrelevant to this review
Walters et al. (1999)	Study design was irrelevant to this review
Wand et al. (2013)	Participants not paediatric (3-18 years)

Wong et al. (2015)	Participants not paediatric (3-18 years)
Yang et al. (2017)	Participants not paediatric (3-18 years)
Yang et al. (2020)	Language: not reported in English, Portuguese, Spanish or German
Zeidan et al. (2016)	Participants not paediatric (3-18 years)
Ziadni et al. (2020)	Study design was irrelevant to this review
Zinman et al. (2005)	Participants not paediatric (3-18 years)

Supplementary Table S4. Summary of mediation analyses using parent-reported outcomes.

Study	Intervention vs Comparator	Path *a* (I→M)	Mediator	Path *b* (M→O)	Outcome *(Parent-Reported)*	Indirect Effect
Lalouni et al. (2020)	Exposure-based internet-CBT vs treatment as usual	NR	GI-specific avoidance behaviour	NR	GI symptoms	+
		NR	GI-specific anxiety	NR	GI symptoms	+
Levy et al. (2014)	SLCBT vs education	+	Perceived pain threat	+	GI symptom severity *at 3 months*	–
		+	Perceived pain threat	+	GI symptom severity *at 6 months*	–
		+	Perceived pain threat	+	GI symptom severity *at 12 months*	+
		+	Perceived pain threat	+	Pain intensity *at 3 months*	+
		+	Perceived pain threat	+	Pain intensity *at 6 months*	+
		+	Perceived pain threat	–	Pain intensity *at 12 months*	+
		+	Solicitousness	–	GI symptom severity *at 3 months*	–
		+	Solicitousness	–	GI symptom severity *at 6 months*	–
		+	Solicitousness	–	GI symptom severity *at 12 months*	–
		+	Solicitousness	–	Pain intensity *at 3 months*	–
		+	Solicitousness	–	Pain intensity *at 6 months*	–
		+	Solicitousness	–	Pain intensity *at 12 months*	–
		+	Pain catastrophizing	–	GI symptom severity *at 3 months*	–
		+	Pain catastrophizing	–	GI symptom severity *at 6 months*	–
		+	Pain catastrophizing	–	GI symptom severity *at 12 months*	–
		+	Pain catastrophizing	–	Pain intensity *at 3 months*	–
		+	Pain catastrophizing	+	Pain intensity *at 6 months*	–
		+	Pain catastrophizing	–	Pain intensity *at 12 months*	–

Statistical significance is defined as 95% confidence intervals that do not contain zero. (+): statistically significant association; (−): statistically non-significant association; CBT, Cognitive Behavioral Therapy; I, Intervention; GI, Gastro-Intestinal; M, Mediator; O, Outcome; SLCBT, Social-Learning Cognitive Behavioral Therapy.

Supplementary Table S5. Results of mediation analyses using parent-reported outcomes.

Study	Intervention vs Comparator → Mediator (Measure) → Outcome (Measure) *Parent-Reported*	Path a Point Estimate (Error)	Path b Point Estimate (Error)	Direct Effect (c′) Point Estimate (Error)	Indirect Effect (ab) Point Estimate (Error)	Total effect (c) Point Estimate (Error)	Proportion Mediated
Lalouni et al. (2021)	Exposure-based internet-CBT vs treatment as usual → GI-avoidance (BRQ-C) → Health-related quality of life (PedQL-GI)	-1.99 (error NR)	-0.72 (error NR)	0.36 (error NR)	1.43 (95% CI=0.42, 3.23)	1.71 (error NR)	79.8%
	Exposure-based internet-CBT vs treatment as usual → GI-anxiety (VSI-C)→ Health-related quality of life (PedQL-GI)	-1.39 (error NR)	-1.13 (error NR)	0.43 (error NR)	1.58 (95% CI=0.43, 3.62)	1.71 (error NR)	78.6%
Levy et al. (2014)	SLCBT vs education → Parent threat (PBQ) → GI symptom severity (CSI) *at 3 months*	-0.18 (SE=0.03; 95% CI=-0.24, -0.12)	0.32 (SE=0.09; 95% CI=0.14, 0.50)	0.01 (SE=0.06; 95% CI= -0.11, 0.13)	-0.06 (SE=0.02; 95% CI=-0.09, 0.02)	NR	NR
	SLCBT vs education → Parent threat (PBQ) → GI symptom severity (CSI) *at 6 months*	-0.18 (SE=0.03; 95% CI=-0.24, -0.12)	0.30 (SE=0.07; 95% CI=0.16, 0.44)	-0.01 (SE=0.05; 95% CI=-0.11, 0.09)	-0.05 (SE=0.02; 95% CI=-0.08, 0.02)	NR	NR
	SLCBT vs education → Parent threat (PBQ) → GI symptom severity (CSI) *at 12 months*	-0.17 (SE=0.03; 95% CI=-0.23, -0.11)	0.33 (SE=0.09; 95% CI=0.15, 0.51)	-0.001 (SE=0.06; 95% CI=-0.12, 0.12	-0.06 (SE=0.02; 95% CI=-0.09, -0.02)	NR	NR
	SLCBT vs education → Parent threat (PBQ) → Pain intensity (FPS-R) *at 3 months*	-0.18 (SE=0.03; 95% CI=-0.24, -0.12)	1.26 (SE=0.27; 95% CI=0.73, 1.79)	-0.02 (SE=0.19; 95% CI=-0.39, 0.35)	-0.23 (SE=0.06; 95% CI=-0.35, -0.11)	NR	NR
	SLCBT vs education → Parent threat (PBQ) → Pain intensity (FPS-R) *at 6 months*	-0.18 (SE=0.03; 95% CI=-0.24, -0.12)	0.88 (SE=0.29; 95% CI=0.31, 1.45)	-0.15 (SE=0.20; 95% CI=-0.54, 0.24)	-0.16 (SE=0.06; 95% CI=-0.27, -0.04)	NR	NR
	SLCBT vs education → Parent threat (PBQ) → Pain intensity (FPS-R) *at 12 months*	-0.18 (SE=0.03; 95% CI=-0.24, -0.12)	0.87 (SE=0.25; 95% CI=-0.38, 1.36)	0.24 (SE=0.18; 95% CI=-0.11, 0.59)	-0.16 (SE=0.05; 95% CI=-0.26, -0.06)	NR	NR
	SLCBT vs education → Parent solicitousness (ARCS) → GI symptom severity (CSI) *at 3 months*	-0.21 (SE=0.03; 95% CI=-0.27, -0.15)	0.09 (SE=0.10; 95% CI=-0.11, 0.29)	0.01 (SE=0.06; 95% CI=-0.11, 0.13)	-0.02 (SE=0.02; 95% CI=-0.06, 0.02)	NR	NR
	SLCBT vs education → Parent solicitousness (ARCS) → GI symptom severity (CSI) *at 6 months*	-0.21 (SE=0.03; 95% CI=-0.26, -0.15)	0.002 (SE=0.09; 95% CI=-0.17, 0.18)	-0.01 (SE=0.05; 95% CI=-0.11, 0.09)	-0.0004 (SE=0.02; 95% CI=-0.04, 0.04)	NR	NR

	SLCBT vs education → Parent solicitousness (ARCS) → GI symptom severity (CSI) *at 12 months*	-0.21 (0.03; 95% CI=-0.27, -0.15)	0.14 (SE=0.11; 95% CI=-0.08, 0.36)	-0.001 (SE=0.06; 95% CI=-0.12, 0.12)	-0.03 (SE=0.02; 95% CI=-0.08, 0.02)	NR	NR
	SLCBT vs education → Parent solicitousness (ARCS) → Pain intensity (FPS-R) *at 3 months*	-0.21 (SE=0.03; 95% CI=-0.27, -0.15)	0.34 (SE=0.32; 95% CI=-0.29, 0.97)	-0.02 (SE=0.19; 95% CI=-0.39, 0.35)	-0.07 (SE=0.07; 95% CI=-0.20, 0.06)	NR	NR
	SLCBT vs education → Parent solicitousness (ARCS) → Pain intensity (FPS-R) *at 6 months*	-0.21 (SE=0.03; 95% CI=-0.27, -0.15)	-0.27 (SE=0.34; 95% CI=-0.94, 0.40)	-0.15 (SE=0.20; 95% CI=-0.54, 0.24)	0.06 (SE=0.07; 95% CI=-0.08, 0.20)	NR	NR
	SLCBT vs education → Parent solicitousness (ARCS) → Pain intensity (FPS-R) *at 12 months*	-0.20 (SE=0.03; 95% CI=-0.26, -0.14)	0.32 (SE=0.30; 95% CI=-0.27, 0.91)	0.24 (SE=0.18; 95% CI=-0.11, 0.59)	-0.06 (SE=0.06; 95% CI=-0.18, 0.06)	NR	NR
	SLCBT vs education → Child catastrophizing (PRI) → GI symptom severity (CSI) *at 3 months*	-0.12 (SE=0.05; 95% CI=-0.22, -0.02)	0.08 (SE=0.07; 95% CI=-0.06, 0.22)	0.01 (SE=0.06; 95% CI= -0.11, 0.13)	-0.01 (SE=0.01; 95% CI=-0.03, 0.01)	NR	NR
	SLCBT vs education → Child catastrophizing (PRI) → GI symptom severity (CSI) *at 6 months*	-0.12 (SE=0.05; 95% CI=-0.22, -0.02)	-0.01 (SE=0.06; 95% CI=-0.13, 0.11)	-0.01 (SE=0.05; 95% CI=-0.11, 0.09)	0.001 (SE=0.01; 95% CI=-0.01, 0.02)	NR	NR
	SLCBT vs education → Child catastrophizing (PRI) → GI symptom severity (CSI) *at 12 months*	-0.12 (SE=0.05; 95% CI=-0.22, -0.02)	0.03 (SE=0.07; 95% CI=-0.11, 0.17)	-0.001 (SE=0.06; 95% CI=-0.12, 0.12)	-0.004 (SE=0.01; 95% CI=-0.02, 0.01)	NR	NR
	SLCBT vs education → Child catastrophizing (PRI) → Pain intensity (FPS-R) *at 3 months*	-0.12 (SE=0.05; 95% CI=-0.22, -0.02)	0.37 (SE=0.21; 95% CI=-0.04, 0.78)	-0.02 (SE=0.19; 95% CI=-0.39, 0.35)	-0.04 (SE=0.03; 95% CI=-0.11, 0.02)	NR	NR
	SLCBT vs education → Child catastrophizing (PRI) → Pain intensity (FPS-R) *at 6 months*	-0.12 (SE=0.05; 95% CI=-0.22, -0.02)	0.03 (SE=0.22; 95% CI=-0.40, 0.46)	-0.15 (SE=0.20; 95% CI=-0.54, 0.24)	-0.0004 (SE=0.03, 95% CI=-0.06, 0.05)	NR	NR
	SLCBT vs education → Child catastrophizing (PRI) → Pain intensity (FPS-R) *at 12 months*	-0.12 (SE=0.05; 95% CI=-0.22, -0.02)	0.25 (SE=0.20; 95% CI=-0.14, 0.64)	0.24 (SE=0.18; 95% CI=-0.11, 0.59)	-0.03 (SE=0.03; 95% CI=-0.08, 0.02)	NR	NR

Supplementary Table S6. Justifications of study quality assessment.

1. PLANNING	Olbrecht et al. (2018)	Bonnert et al. (2018)	Lalouni et al. (2020)	Levy et al. (2014)	Kashikar-Zuck et al. (2013)	Wicksell et al. (2011)
1.1 Was the mediation analyses planned a priori in the trial protocol?	No	Yes (NCT02306369)	Yes (NCT02873078)	No	No	No
1.2 Was the choice of mediators based on clinical rationale underlying the mechanisms through which the treatment affects the outcome, or based on independent data?	Yes, based on clinical rationale.	Yes, based on clinical rationale and independent data.	Yes, based on clinical rationale and independent data.	Yes, based on clinical rationale and independent data.	Yes, based on clinical rationale and independent data.	Yes, based on clinical rationale and independent data.
1.3[a] Was there a plan to collect pre- and post-randomization confounders of the mediator-mediator and mediator-outcome relationships? Could the authors foresee if any of these confounders were treatment-induced (e.g. collected after the onset of treatment, and therefore possibly affected by treatment)?	Yes, there was a plan to collect data on variables used to calculate propensity scores, to use for all linear models, thus adjusting for exposure-mediator, mediator-outcome and exposure-outcome relationships. No discussion of potential that confounders were treatment-induced.<br><br>n/a - mediator-mediator confounders	No	No	No	No	No
1.4 Were the mediators measured prior to the outcome, and preferably repeatedly, to assure	Yes, the mediators were measured prior to the outcome.	Yes, the mediators were measured prior to the outcome.	Yes, the mediators were measured prior to the outcome.	Yes, the mediators were measured prior to the outcome.	Yes, the mediators were measured prior to the outcome.	Yes, the mediators were measured prior to the outcome.

the causal interpretation of the findings?	No, the mediator was not measured at repeated time points	Yes, the mediators were measured at repeated time points	Yes, the mediators were measured at repeated time points	No, the mediator was not measured at repeated time points	No, the mediator was not measured at repeated time points.	No, the mediator was not measured at multiple time points.
1.5 Was a causal diagram reported, underlying the causal relationship of the treatment, mediator(s) and outcome?	Yes	Yes	Yes	Yes	No	No
1.6 Was the sample size for the mediation analysis estimated?	No	No	No	No	No	No
1.7 Was the conduct of a mediation analysis dependent on whether a statistically significant intention-to-treat treatment effect was found?	Unsure<br><br>There was a difference in outcome between groups, but it is not clear this was known prior to the analysis (primary data), and there is no mention that the analysis was *dependent* on a treatment effect being present.	Unsure<br><br>It is stated that a treatment effect in the primary RCT was found. It is not stated that mediation analysis was *dependent* on treatment effect.	Unsure<br><br>It is stated that a treatment effect in the primary RCT was found. It is not stated that mediation analysis was *dependent* on treatment effect. Mediation analysis was pre-planned in the trial protocol with no stipulation of the necessity of a treatment effect to proceed with mediation analysis.	Unsure<br><br>It is not explicitly stated that mediation analysis was *dependent* on treatment effect. However, it is stated that in the primary RCT that a significant treatment effect is present, and an analysis is conducted demonstrating that the putative mediators all had a moderate-large effect size change from baseline to post-treatment compared between groups.	Unsure<br><br>It is not explicitly stated that conduct of mediation was *dependent* on treatment effect, however outcomes (depression, disability) were chosen for mediation analysis because these were significant in the primary RCT, and others (e.g., pain intensity) were not.	Unsure<br><br>It is stated that a treatment effect was found. It is not stated that the conduct of the mediation was *dependent* on a treatment effect being present.
**2. CONDUCT**						

2.1 Was multiple imputation (or other valid approaches) used to handle missing data? If a complete-case analysis was used, did they adjust for baseline covariates that were differentially distributed between responders and non-responders? Was a sensitivity analysis conducted to assess the impact of different approaches on the findings?	Missing data handled using list-wise deletion<br><br>Yes, a complete-case analysis was conducted. Yes, there was adjustment for baseline covariates using propensity scores.<br><br>No sensitivity analysis.	Missing data was assumed missing at random and handled using full information maximum likelihood.<br><br>No, a complete-case analysis was not used.<br><br>No sensitivity analysis.	Missing data was handled using full information maximum likelihood.<br><br>No, a complete-case analysis was not used.<br><br>No sensitivity analysis.	Missing data was handled using full information maximum likelihood.<br><br>No, a complete-case analysis was not used.<br><br>No sensitivity analysis.	No missing data.<br><br>Yes, a complete-case analysis was conducted. No adjustment for baseline covariates as no significant differences between groups for measured variables.<br><br>n/a – sensitivity analysis.	No imputation was used.<br><br>Unclear if a complete-case analysis was conducted. No reporting of adjusting for baseline covariates.<br><br>No sensitivity analysis.
2.2 Does the study report separate analyses for separate mediators?	n/a – one mediator tested	Yes	Yes	Yes	Yes	Yes
2.3 Does the study use an appropriate framework for analysis?	Yes, product of coefficient approach justified assuming both models for the outcome and mediator are linear with no interaction.	Yes, product of coefficient approach justified assuming both models for the outcome and mediator are linear with no interaction.	Yes, product of coefficient approach justified assuming both models for the outcome and mediator are linear with no interaction.	Yes, structural equation modelling approach justified assuming both models for the outcome and mediator are linear with no interaction.	Yes, product of coefficient approach justified assuming both models for the outcome and mediator are linear with no interaction.	Yes, product of coefficient approach justified assuming both models for the outcome and mediator are linear with no interaction.
2.4 Does the study assess potential interaction(s) between treatment and confounding factors, treatment and mediator, mediator and mediator in the mediator and outcome models? Does the study evaluate the goodness-of-fit of each model?	No assessment of potential interaction(s)<br><br>No goodness-of-fit model.	No assessment of potential interaction(s).<br><br>No goodness-of-fit model.	Yes, the study assesses potential interactions between the treatment and mediator.<br><br>No goodness-of-fit model.	No assessment of potential interaction(s)<br><br>No goodness-of-fit model.	No assessment of potential interaction(s)<br><br>No goodness-of-fit model.	No assessment of potential interaction(s)<br><br>No goodness-of-fit model.

2.5[a] Does the study adjust for mediator-mediator and mediator-outcome confounders?	Mediator-mediator confounders – n/a<br><br>Yes, adjusts for both exposure-mediator and mediator-outcome confounders. The study adjusts for baseline covariates by using propensity scores in all analyses.	No adjustment for mediator-mediator confounders<br><br>Yes, adjusted for mediator-outcome confounders by adjusting for reciprocal mediators in the multiple mediator model.	No adjustment for mediator-mediator or mediator-outcome confounders.	No adjustment for mediator-mediator confounders<br><br>Yes, adjusted for mediator-outcome confounders by adjusting for reciprocal mediators in the multiple mediator model.	No adjustment for mediator-mediator or mediator-outcome confounders.	No adjustment for mediator-mediator or mediator-outcome confounders.
2.6 Does the study perform sensitivity analysis to assess sensitivity of the results to (1) the assumption of no measured mediator-mediator or mediator-outcome confounders, (2) potential measurement errors of the mediators?	No sensitivity analyses	No sensitivity analyses	No sensitivity analyses	No sensitivity analyses	No sensitivity analyses	No sensitivity analyses
2.7 Does the study use apt strategies when some of the mediator-mediator or mediator-outcome confounders are potentially affected by the treatment (e.g., by considering confounders as mediators themselves)?	No	No	No	No	No	No
**3. REPORTING**						
3.1 Does the study report the approaches used for mediation and provide a causal diagram that underlies the analysis?	Yes, the approach for mediation is described, and a causal diagram is provided.	Yes, the approach for mediation is described, and a causal diagram is provided.	Yes, the approach for mediation is described, and a causal diagram is provided.	Yes, the approach for mediation is described, and a causal diagram is provided.	Yes, the approach for mediation is described.<br>No, a causal diagram is not provided.	Yes, the approach for mediation is described.<br>No, a causal diagram is not provided.

3.2 Does the study report the sample size calculation, the actual sample size of the mediation analysis and how the missing data is handled?	No sample size calculation provided.<br><br>Yes, actual sample size is described.<br><br>Yes, description of how missing data were handled.	No sample size calculation provided.<br><br>Yes, actual sample size is described.<br><br>Yes, description of how missing data were handled.	No sample size calculation provided.<br><br>Yes, actual sample size is described.<br><br>Yes, description of how missing data were handled.	No sample size calculation provided.<br><br>Yes, actual sample size is described.<br><br>Yes, description of how missing data were handled.	No sample size calculation provided.<br><br>Yes, described actual sample size.<br><br>n/a – no missing data.	No sample size calculation provided.<br><br>Yes, described actual sample size.<br><br>No description of how missing data were handled.
3.3 Does the study report all confounders considered and adjusted for in the analysis?	Yes, all baseline covariates considered and adjusted for via propensity scores are reported.	No confounders were considered or adjusted for.	No confounders were considered or adjusted for.	Yes, baseline values of the mediator and outcome variables that were considered and adjusted for in the analysis as covariates are reported.	No confounders were considered or adjusted for.	No confounders were considered or adjusted for.
3.4 Does the study report the model building procedure and the final form of all models used in the analysis? Do they report the goodness-of-fit of these models?	No, model building procedures and final form of all models in the analysis are not described.<br><br>No goodness-of-fit metric is provided.	No, model building procedures and final form of all models in the analysis are not described.<br><br>No goodness-of-fit metric is provided.	No, model building procedures and final form of all models in the analysis are not described.<br><br>No goodness-of-fit metric is provided.	No, model building procedures and final form of all models in the analysis are not described.<br><br>No goodness-of-fit metric is provided.	No, model building procedures and final form of all models in the analysis are not described.<br><br>No goodness-of-fit metric is provided.	No, model building procedures and final form of all models in the analysis are not described.<br><br>No goodness-of-fit metric is provided.
3.5 Does the study report the point estimates and the confidence intervals (CIs) of the different direct, indirect and total treatment effects?	Reported: point estimates of the direct and total effect.<br><br>Not reported: point estimates of the indirect effect, and CIs of direct, indirect or total effect.	Reported: point estimates and CIs of the indirect and direct effect.<br><br>Not reported: point estimates and CIs of the total effect.	Reported: point estimates and CIs of the indirect effect.<br><br>Not reported: point estimates and CIs of the direct or total effect.	Reported: point estimates and CIs of the indirect and direct effect.<br><br>Not reported: point estimates and CIs of the total effect.	Reported: point estimates and CIs of the indirect effect.<br><br>Not reported: point estimate and CIs of the direct and total effects.	Reported: point estimates for the direct, indirect and total effects and CIs of the indirect effect.<br><br>Not reported: CIs of the direct and total effects.

3.6 Does the study report the methods and results of all sensitivity and other additional analyses (in the main paper or appendices)?	N/a - no sensitivity or other analyses are conducted.	Yes, the study reports methods and results of additional analyses (time-lagged analysis). n/a for sensitivity analysis as none conducted.	N/a - no sensitivity or other analyses are conducted.	N/a - no sensitivity or other analyses are conducted.	N/a - no sensitivity or other analyses are conducted.	Yes, the study reports methods and results of additional analyses.<br><br>n/a for sensitivity analysis as none conducted.
3.7 Does the study discuss the validity of all causal assumptions underlying the analysis (in the main paper or appendices)?	No - Does not discuss assumption of temporal ordering.<br><br>Yes - Acknowledges the plausibility of bias due to unmeasured confounders. But, does not justify all measured confounders are adequate to fully adjust for confounding. Also does not follow with a sensitivity analysis to test the plausibility of the assumption.	Yes - Acknowledges the requirement for temporal ordering and conducts a time-lagged analyses to justify the validity of the assumption of temporal ordering<br><br>No - Does not acknowledge or justify the plausibility of the assumption of no unmeasured confounding. Does not follow with a sensitivity analysis to test the plausibility of the assumption.	Yes - Acknowledges the limitation that temporal order between mediator and outcome was not established. Does not conduct analyses to justify the validity of the assumption of temporal ordering.<br><br>Yes - Acknowledges the limitation that confounders were not measured or assessed. Does not conduct a sensitivity analysis to test the plausibility of the assumption of no unmeasured confounders.	Yes - Acknowledges the requirement for temporal ordering and conducts a time-lagged mediation model to justify the validity of the assumption of temporal ordering<br><br>No - Does not acknowledge or justify the plausibility of the assumption of no unmeasured confounding. Does not follow with a sensitivity analysis to test the plausibility of the assumption.	Yes - Acknowledges the requirement for temporal ordering. Does not conduct analyses to justify the validity of the assumption of temporal ordering.<br><br>No - Does not acknowledge or justify the plausibility of the assumption of no unmeasured confounding. Does not follow with a sensitivity analysis to test the plausibility of the assumption.	Yes - Acknowledges the requirement for temporal ordering and conducts hierarchical regression analyses to justify the validity of the assumption of temporal ordering.<br><br>No - Does not acknowledge or justify the plausibility of the assumption of no unmeasured confounding. Does not follow with a sensitivity analysis to test the plausibility of the assumption.

[a]At item 1.3 and 2.5, for observational study designs we also considered adjustment for intervention-mediator and intervention-outcome confounders.

Supplementary Table S7. Findings of mediation analyses of included studies (with child-reported outcomes).

Study	Intervention vs Comparator → Mediator (Measure) → Outcome (Measure) *Child-Reported*	Path a Point Estimate (Error)	Path b Point Estimate (Error)	Direct effect (c′) Point Estimate (Error)	Indirect Effect (ab) Point Estimate (error)	Total Effect (c) Point Estimate (Error)	Proportion Mediated
Olbrecht et al. (2018)	Intravenous acetaminophen vs no intravenous acetaminophen → Morphine consumption → Hospital length of stay	-0.575 (SE=0.133; 95% CI=-0.84, -0.31; p<0.0001)	0.539 (SE=0.178; 95% CI=0.19, 0.89; p<0.0032)	-0.077 (SE=0.262; 95% CI=-0.59, 0.44; p=0.770)	-0.31 (Sobel's test p=0.013)	-0.361 (SE=0.253; 95% CI=-0.86, 0.13; p=0.157)	78.72%
Bonnert et al. (2018)	Exposure-based internet-CBT vs waitlist → Avoidance behavior (IBS-BRQ)→ GI symptoms (GSRS-IBS)	-1.08 (SE=0.39; 95% CI=-1.84, -0.32; p=0.0060	0.34 (SE=0.02; 95% CI=0.30, 0.38; p<0.001)	−0.17 (SE=0.19; 95% CI=-0.54, 0.20; p=0.373)	−0.37 (95% CI= -0.62, -0.09)	NR	67.3%
	Exposure-based internet-CBT vs waitlist→ Perceived stress (PSS-10) → GI symptoms (GSRS-IBS)	0.004 (SE=0.11; 95% CI=-0.21, 0.22; p=0.972)	0.41 (SE=0.06, 95% CI=0.29, 0.53; p<0.001)	−0.55 (SE=0.21; 95% CI=-0.96, -0.14, p=0.009)	0.002 (95% CI = −0.08, 0.09)	NR	0.3%
Lalouni et al. (2021)	Exposure-based internet-CBT vs treatment as usual → GI-avoidance (BRQ-C) → Health-related quality of life (PedQL-GI)	-2.14 (SE=0.71; 95% CI=-3.54, -0.75; p=0.003)	-0.81 (SE=0.24; 95% CI=-1.27, -0.35; p=0.001)	NR	1.73 (95% CI=0.48, 3.64)	NR	NR
	Exposure-based internet-CBT vs treatment as usual → GI-anxiety (VSI-C)→ Health-related quality of life (PedQL-GI)	-1.38 (SE=0.33; 95% CI=-2.03, -0.73; p=0.012)	-1.62 (SE=0.33; 95% CI=-2.27, -0.97; p<0.001)	NR	2.23 (95% CI=0.66, 4.37)	NR	NR
Levy et al. (2014)	SLCBT vs education → Parent threat (PBQ) → GI symptom severity (CSI) *at 3 months*	-0.17 (SE=0.03; 95% CI=-0.23, -0.11)	0.06 (SE=0.07; 95% CI=-0.08, 0.20)	0.02 (SE=0.05; 95% CI= -0.08, 0.12)	-0.01 (SE=0.01; 95% CI=-0.03, 0.01)	NR	NR
	SLCBT vs education → Parent threat (PBQ) → GI symptom severity (CSI) *at 6 months*	-0.18 (SE=0.03; 95% CI=-0.24, -0.12)	0.13 (SE=0.07; 95% CI=-0.01, 0.27)	-0.003 (SE=0.05; 95% CI=-0.10, 0.10)	-0.02 (SE=0.01; 95% CI=-0.05, 0.002)	NR	NR
	SLCBT vs education → Parent threat (PBQ) → GI symptom severity (CSI) *at 12 months*	-0.18 (SE=0.03; 95% CI=-0.24, -0.12)	0.05 (SE=0.09; 95% CI=-0.13, 0.23)	-0.11 (SE=0.06; 95% CI=-0.23, 0.01	-0.01 (SE=0.02; 95% CI=-0.04, 0.02)	NR	NR

	SLCBT vs education → Parent threat (PBQ) → Pain intensity (FPS-R) *at 3 months*	-0.18 (SE=0.03; 95% CI=-0.24, -0.12)	0.59 (SE=0.19; 95% CI=0.22, 0.96)	0.03 (SE=0.13; 95% CI=-0.22, 0.28)	-0.11 (SE=0.04; 95% CI=-0.18, -0.03)	NR	NR
	SLCBT vs education → Parent threat (PBQ) → Pain intensity (FPS-R) *at 6 months*	-0.18 (SE=0.03; 95% CI=-0.24, -0.12)	0.37 (SE=0.17; 95% CI=0.04, 0.70)	0.06 (SE=0.12; 95% CI=-0.18, 0.30)	-0.07 (SE=0.03; 95% CI=-0.13, -0.003)	NR	NR
	SLCBT vs education → Parent threat (PBQ) → Pain intensity (FPS-R) *at 12 months*	-0.17 (SE=0.03; 95% CI=-0.23, -0.11)	0.28 (SE=0.19; 95% CI=-0.09, 0.65)	0.09 (SE=0.13; 95% CI=-0.16, 0.34)	-0.05 (SE=0.03; 95% CI=-0.11, 0.02)	NR	NR
	SLCBT vs education → Parent solicitousness (ARCS) → GI symptom severity (CSI) *at 3 months*	-0.21 (SE=0.03; 95% CI=-0.27, -0.15)	-0.01 (SE=0.08; 95% CI=-0.17, 0.15)	0.02 (SE=0.05; 95% CI=-0.08, 0.12)	0.002 (SE=0.02; 95% CI=-0.03, 0.03)	NR	NR
	SLCBT vs education → Parent solicitousness (ARCS) → GI symptom severity (CSI) *at 6 months*	-0.21 (SE=0.03; 95% CI=-0.27, -0.15)	-0.08 (SE=0.08; 95% CI=-0.24, 0.08)	-0.003 (SE=0.05; 95% CI=-0.10, 0.10)	0.02 (SE=0.02; 95% CI=-0.02, 0.05)	NR	NR
	SLCBT vs education → Parent solicitousness (ARCS) → GI symptom severity (CSI) *at 12 months*	-0.21 (0.03; 95% CI=-0.27, -0.15)	-0.08 (SE=0.10; 95% CI=-0.28, 0.12)	-0.11 (SE=0.06; 95% CI=-0.23, 0.01)	0.02 (SE=0.02; 95% CI=-0.02, 0.06)	NR	NR
	SLCBT vs education → Parent solicitousness (ARCS) → Pain intensity (FPS-R) *at 3 months*	-0.21 (SE=0.03; 95% CI=-0.27, -0.15)	-0.16 (SE=0.23; 95% CI=-0.61, 0.29)	0.03 (SE=0.13; 95% CI=-0.22, 0.28)	0.03 (SE=0.05; 95% CI=-0.06, 0.13)	NR	NR
	SLCBT vs education → Parent solicitousness (ARCS) → Pain intensity (FPS-R) *at 6 months*	-0.21 (SE=0.03; 95% CI=-0.27, -0.15)	-0.37 (SE=0.20; 95% CI=-0.76, 0.02)	0.06 (SE=0.12; 95% CI=-0.18, 0.30)	0.08 (SE=0.04; 95% CI=-0.01, 0.16)	NR	NR
	SLCBT vs education → Parent solicitousness (ARCS) → Pain intensity (FPS-R) *at 12 months*	-0.21 (SE=0.03; 95% CI=-0.27, -0.15)	-0.10 (SE=0.23; 95% CI=-0.55, 0.35)	0.09 (SE=0.13; 95% CI=-0.16, 0.34)	0.02 (SE=0.05; 95% CI=-0.07, 0.12)	NR	NR
	SLCBT vs education → Child catastrophizing (PRI) → GI symptom severity (CSI) *at 3 months*	-0.12 (SE=0.05; 95% CI=-0.22, -0.02)	0.14 (SE=0.05; 95% CI=0.04, 0.24)	0.02 (SE=0.05; 95% CI= -0.08, 0.12)	-0.02 (SE=0.01; 95% CI=-0.03, -0.001)	NR	NR
	SLCBT vs education → Child catastrophizing (PRI) → GI symptom severity (CSI) *at 6 months*	-0.12 (SE=0.05; 95% CI=-0.22, -0.02)	0.18 (SE=0.05; 95% CI=0.08, 0.28)	-0.003 (SE=0.05; 95% CI=-0.10, 0.10)	-0.02 (SE=0.01; 95% CI=-0.04, 0.0004)	NR	NR
	SLCBT vs education → Child catastrophizing (PRI) → GI symptom severity (CSI) *at 12 months*	-0.12 (SE=0.05; 95% CI=-0.22, -0.02)	0.12 (SE=0.07; 95% CI=-0.02, 0.26)	-0.11 (SE=0.06; 95% CI=-0.23, 0.01)	-0.01 (SE=0.01; 95% CI=-0.03, 0.06)	NR	NR

	SLCBT vs education → Child catastrophizing (PRI) → Pain intensity (FPS-R) *at 3 months*	-0.12 (SE=0.05; 95% CI=-0.22, 0.02)	0.24 (SE=0.15; 95% CI=-0.05, 0.53)	0.03 (SE=0.13; 95% CI=-0.22, 0.28)	-0.03 (SE=0.02; 95% CI=-0.07, 0.01)	NR	NR
	SLCBT vs education → Child catastrophizing (PRI) → Pain intensity (FPS-R) *at 6 months*	-0.12 (SE=0.05; 95% CI=-0.22, 0.02)	0.26 (SE=0.13; 95% CI=0.005, 0.52)	0.06 (SE=0.12; 95% CI=-0.18, 0.30)	-0.03 (SE=0.02, 95% CI=-0.07, 0.009)	NR	NR
	SLCBT vs education → Child catastrophizing (PRI) → Pain intensity (FPS-R) *at 12 months*	-0.12 (SE=0.05; 95% CI=-0.22, 0.02)	0.16 (SE=0.15; 95% CI=-0.13, 0.45)	0.09 (SE=0.13; 95% CI=-0.16, 0.34)	-0.02 (SE=0.02; 95% CI=-0.06, 0.02)	NR	NR
Kashikar-Zuck et al. (2013)	CBT vs fibromyalgia education → Pain coping (PCQ) → Functional disability (FDI) *at 6 months*	NR	NR	NR	−0.004 (95% CI = −0.06, 0.06)	NR	NR
	CBT vs fibromyalgia education → Pain coping (PCQ) → Depression (CDI) *at 6 months*	NR	NR	NR	−0.12 (95% CI = −0.82, 0.46)	NR	NR
	CBT vs fibromyalgia education → Catastrophizing (PCQ) → Functional disability (FDI) *at 6 months*	NR	NR	NR	0.02 (95% CI = −0.04, 0.09)	NR	NR
	CBT vs fibromyalgia education → Catastrophizing (PCQ) → Depression (CDI) *at 6 months*	NR	NR	NR	−0.32 (95% CI = −1.43, 0.19)	NR	NR
	CBT vs fibromyalgia education → Coping efficacy (PCQ) → Functional disability (FDI) *at 6 months*	NR	NR	NR	−0.04 (95% CI = −0.10, 0.03)	NR	NR
	CBT vs fibromyalgia education → Coping efficacy (PCQ) → Depression (CDI) *at 6 months*	NR	NR	NR	−0.24 (95% CI = −1.15, 0.38)	NR	NR
Wicksell et al. (2011)	ACT vs MDT + amitriptyline → Pain impairment beliefs (PAIRS) → Pain interference (PII) *at 3.5 months*	16.18 (SE=5.92; 95% CI=4.58, 27.78; p=0.0121)	0.05 (SE=0.04; 95% CI=-0.03, 0.13; p=0.2607	-0.21 (SE=1.42, 95% CI=-2.99, 2.57; p=0.8847)	0.83 (SE=0.74; 90% CI=0.00, 2.40; 95% CI=-0.62, 2.28; p=0.2669)	0.62 (SE=1.23; 95% CI=-1.79, 3.03; p=0.6218)	NR
	ACT vs MDT + amitriptyline → Pain impairment beliefs (PAIRS) → Pain interference (PII) *at 7 months*	13.83 (SE=4.86; 95% CI=4.30, 23.36; p=0.0103)	0.08 (SE=0.07; 95% CI=-0.06, 0.22; p=0.2148)	0.40 (SE=1.66; 95% CI=-2.85, 3.65; p=0.8115)	1.16 (SE=0.95; 90% CI=-0.41, 3.18; 95% CI=-0.70, 3.02; p=0.2188)	1.57 (SE=1.41; 95% CI=-1.19, 4.33; p=0.2807)	NR
	ACT vs MDT + amitriptyline → Pain impairment beliefs (PAIRS) → Depression (CES-DC) *at 3.5 months*	16.02 (SE=5.69; 95% CI=4.87, 27.17; p=0.0099)	0.43 (SE=0.019; 95% CI=0.39, 0.47; p=0.0358)	-0.31 (SE=6.09; 95% CI=-12.25, 11.63; p=0.9597)	6.89 (SE=3.80; 95% CI=1.75, 14.59; p=0.0699)	6.58 (SE=5.69; 95% CI=-4.57,	NR

						17.73; p=0.2595)	
	ACT vs MDT + amitriptyline → Pain impairment beliefs (PAIRS) → Depression (CES-DC) *at 7 months*	17.64 (SE=5.43; 95% CI=7.00, 28.28; p=0.0047)	0.66 (SE=0.33; 95% CI=0.01, 1.31; p=0.0662)	-4.36 (SE=9.48; 95% CI=-22.94, 14.22; p=0.6520)	11.56 (SE=6.52; 95% CI=2.46, 26.55; p=0.0763)	7.20 (SE=8.05; 95% CI=-8.58, 22.98; p=0.3835)	NR
	ACT vs MDT + amitriptyline → Pain reactivity (PRS) → Pain interference (PII) *at 3.5 months*	1.80 (SE=0.88; 95% CI=0.08, 3.52; p=0.0535)	0.61 (SE=0.31; 95% CI=0.01, 1.22; p=0.0617)	0.03 (SE=1.32; 95% CI=-2.56, 2.62; p=0.9846)	1.10 (SE=0.74; 95% CI=0.08, 3.01; p=0.1384)	1.12 (SE=1.29; 95% CI=-1.41, 3.65; p=0.3941)	NR
	ACT vs MDT + amitriptyline → Pain reactivity (PRS) → Pain interference (PII) *at 7 months*	1.74 (SE=0.87; 95% CI=0.03, 3.45; p=0.0617)	0.97 (SE=0.32; 95% CI=0.34, 1.60; p=0.0075)	0.29 (SE=1.31; 95% CI=-2.28, 2.86; p=0.8281)	1.69 (SE=0.98; 95% CI=0.17, 4.32; p=0.844)	1.98 (SE=1.43; 95% CI=-0.82, 4.78; p=0.1836)	NR
	ACT vs MDT + amitriptyline → Pain reactivity (PRS) → Depression (CES-DC) *at 3.5 months*	1.98 (SE=0.87; 95% CI=0.27, 3.69; p=0.0329)	2.75 (SE=1.33; 95% CI=0.14, 5.36; p=0.0519)	4.52 (SE=5.89; 95% CI=-7.02, 16.06; p=0.4510)	5.43 (SE=3.41; 95% CI=0.01, 14.77; p=0.1119)	9.95 (SE=5.66; 95% CI=-1.14, 21.04; p=0.935)	NR
	ACT vs MDT + amitriptyline → Pain reactivity (PRS) → Depression (CES-DC) *at 7 months*	1.45 (SE=0.92; 95% CI=-0.35, 3.25; p=0.1330)	4.16 (SE=2.03; 95% CI=0.18, 8.14; p=0.0582)	4.26 (SE=8.02; 95% CI=-11.46, 19.98; p=0.6028)	6.05 (SE=4.63; 90% CI=3.52, 31.96; 95% CI=-3.02, 15.12; p=0.1912)	10.31 (SE=8.17; 95% CI=-5.70, 26.32; p=0.2248)	NR
	ACT vs MDT + amitriptyline → Self-efficacy (SES) → Pain interference (PII) *at 3.5 months*	-23.31 (SE=12.70; 95% CI=-48.20, 1.58; p=0.0806)	-0.02 (SE=0.02; 95% CI=-0.06, 0.02; p=0.4008)	0.08 (SE=1.40; 95% CI=-2.66, 2.82; p=0.9572)	0.48 (SE=0.55; 90% CI=-0.35, 1.79; 95% CI=-0.60, 1.56; p=0.4168)	0.52 (SE=1.30; 95% CI=-2.03, 3.07; p=0.6896)	NR
	ACT vs MDT + amitriptyline → Self-efficacy (SES) → Pain interference (PII) *at 7 months*	-25.98 (SE=14.89; 95% CI=-55.16, 3.20; p=0.0981)	-0.02 (SE=0.02; 95% CI=-0.06, 0.02; p=0.4566)	0.99 (SE=1.63; 95% CI=-2.20, 4.18; p=0.5524)	0.47 (SE=0.64; 90% CI=-0.27, 2.32; 95% CI=-0.78, 1.72; p=0.4626)	1.45 (SE=1.49; 95% CI=-1.47, 4.37; p=0.3400)	NR
	ACT vs MDT + amitriptyline → Self-efficacy (SES) → Depression (CES-DC) *at 3.5 months*	-23.92 (SE=12.23; 95% CI=-47.89, 0.06; p=0.0634)	-0.04 (SE=0.11; 95% CI=-0.26, 0.18; p=0.6946)	5.35 (SE=6.60; 95% CI=-7.56, 18.29; p=0.4264)	1.01 (SE=2.48; 90% CI -3.28, 7.42; 95% CI=-3.85, 5.87; p=0.6834)	6.36 (SE=5.97; 95% CI=-5.34, 18.06; p=0.2983)	NR
	ACT vs MDT + amitriptyline → Self-efficacy (SES) → Depression (CES-DC) *at 7 months*	-21.03 (SE=15.24; 95% CI=-50.90, 8.84; p=0.1865)	0.02 (SE=0.15; 95% CI=-0.27, 0.31; p=0.9168)	7.35 (SE=9.43; 95% CI=-11.13, 25.83; p=0.4477)	-0.33 (SE=2.90; 90% CI=-10.22, 3.09; 95% CI=-6.01, 5.35; p=0.9102)	7.03 (SE=8.64; 95% CI=-9.90, 23.96; p=0.4278)	NR

	ACT vs MDT + amitriptyline → Kinesiophobia (TSK) → Pain interference (PII) *at 3.5 months*	3.84 (SE=3.03; 95% CI=-2.10, 9.78; p=0.2172)	0.07 (SE=0.09; 95% CI=-0.11, 0.25; p=0.4513)	0.36 (SE=1.29; 95% CI=-2.17, 2.89; p=0.7839)	0.26 (SE=0.38; 90% CI=-0.17; 95% CI=-0.48, 1.00; 1.43, p=0.4945)	0.62 (SE=1.23; 95% CI=-1.79, 3.03; p=0.6218)	NR
	ACT vs MDT + amitriptyline → Kinesiophobia (TSK) → Pain interference (PII) *at 7 months*	3.78 (SE=2.92; 95% CI=-1.94, 9.50; p=0.2119)	0.19 (SE=0.10; 95% CI=-0.01, 0.39; p=0.0879)	0.85 (SE=1.39; 95% CI=-1.87, 3.57; p=0.55)	0.71 (SE=0.66; 90% CI=-0.15, 2.62; 95% CI=-0.58, 2.00; p=0.2768)	1.57 (SE=1.41; 95% CI=-1.19, 4.33; p=0.2807)	NR
	ACT vs MDT + amitriptyline → Kinesiophobia (TSK) → Depression (CES-DC) *at 3.5 months*	4.00 (SE=2.92; 95% CI=-1.72, 9.72; p=0.1836)	0.36 (SE=0.41; 95% CI=-0.44, 1.16; p=0.3895)	5.14 (SE=5.95; 95% CI=-6.52, 16.80; p=0.3965)	1.44 (SE=1.87; 90% CI=-0.85, 7.76; 95% CI=-2.23, 5.11; p=0.4430)	6.58 (SE=5.69; 95% CI=-4.57, 17.73; p=0.2595)	NR
	ACT vs MDT + amitriptyline → Kinesiophobia (TSK) → Depression (CES-DC) *at 7 months*	4.33 (SE=3.47; 95% CI=-2.47, 11.13; p=0.2285)	0.59 (SE=0.56; 95% CI=-0.51, 1.68; p=0.3065)	4.64 (SE=8.39; 95% CI=-11.80, 21.08; p=0.5880)	2.57 (SE=3.04; 90% CI=-1.29, 16.77; 95% CI=-3.39, 8.53; p=0.3984)	7.20 (SE=8.05; 95% CI=-8.58, 22.98; p=0.3835)	NR
	ACT vs MDT + amitriptyline → Catastrophizing (PCQ) → Pain interference (PII) *at 3.5 months*	-1.39 (SE=1.89; 95% CI=-5.09, 2.31; p=0.4710)	0.08 (SE=0.15; 95% CI=-0.21, 0.37; p=0.5956)	1.10 (SE=1.28; 95% CI=-1.41, 3.61; p=0.4000)	-0.11 (SE=0.24; 90% CI=-1.04, 0.09; 95% CI=-0.58, 0.36; p=0.6513)	0.99 (SE=1.25; 95% CI=-1.46, 3.44; p=0.4337)	NR
	ACT vs MDT + amitriptyline → Catastrophizing (PCQ) → Pain interference (PII) *at 7 months*	-2.28 (SE=1.94; 95% CI=-6.08, 1.52; p=0.2554)	-0.03 (SE=0.18; 95% CI=-0.38, 0.32; p=0.8843)	1.92 (SE=1.53; 95% CI=-1.08, 4.92; p=0.2259)	0.06 (SE=0.39; 90% CI=-0.47, 0.74; 95% CI=-0.70, 0.82; p=0.88)	1.98 (SE=1.43; 95% CI=-0.82, 4.78; p=0.1836)	NR
	ACT vs MDT + amitriptyline → Catastrophizing (PCQ) → Depression (CES-DC) *at 3.5 months*	-0.84 (SE=1.94; 95% CI=-4.64, 2.96; p=0.6678)	0.51 (SE=0.63; 95% CI=-0.72, 1.74; p=0.4295)	9.09 (SE=5.73; 95% CI=-2.14, 20.32; p=0.1274)	-0.43 (SE=1.08; 90% CI=-4.73, 0.90; 95% CI=-2.55, 1.69; p=0.6940)	8.66 (SE=5.66; 95% CI=-2.43, 19.75; p=0.1399)	NR
	ACT vs MDT + amitriptyline → Catastrophizing (PCQ) → Depression (CES-DC) *at 7 months*	-2.22 (SE=1.98; 95% CI=-6.10, 1.66; p=0.2795)	0.25 (SE=1.06; 95% CI=-1.83, 2.33; p=0.8179)	10.86 (SE=8.74; 95% CI=-6.27, 27.99; p=0.2331)	-0.55 (SE=2.26; 90% CI=-11.14, 0.44; 95% CI=-4.98, 3.88; p=0.8074)	10.31 (SE=8.17; 95% CI=-5.70, 26.32; p=0.2248)	NR
	ACT vs MDT + amitriptyline → Pain intensity (VAS) → Pain interference (PII) *at 3.5 months*	1.41 (SE=1.05; 95% CI=-0.65, 3.47; p=0.1920)	0.42 (SE=0.24; 95% CI=-0.05, 0.89; p=0.0921)	0.02 (SE=1.23; 95% CI=-2.39, 2.43; p=0.9869)	0.60 (SE=0.54; 90% CI=-0.02, 2.18; 95% CI=-0.46, 1.66; p= 0.2697)	0.62 (SE=1.23; 95% CI=-1.79, 3.03; p=0.6218)	NR
	ACT vs MDT + amitriptyline → Pain intensity (VAS) → Pain interference (PII) *at 7 months*	1.64 (SE=1.11; 95% CI=-0.54, 3.82; p=0.1564)	0.32 (SE=0.29; 95% CI=-0.25, 0.89; p=0.2845)	1.04 (SE=1.48; 95% CI=-1.86, 3.94; p=0.4905)	0.52 (SE=0.57; 90% CI=-0.04, 2.35; 95% CI=-0.60, 1.64; p=0.3564)	1.57 (SE=1.41; 95% CI=-1.19, 4.33; p=0.2807)	NR

	ACT vs MDT + amitriptyline → Pain intensity (VAS) → Depression (CES-DC) *at 3.5 months*	1.30 (SE=1.02; 95% CI=-0.70, 3.30; p=0.2162)	0.60 (SE=1.18; 95% CI=-1.71, 2.91; p=0.6149)	5.80 (SE=5.98; 95% CI=-5.92, 17.52, p=0.3432)	0.78 (SE=1.59; 90% CI=-1.33, 6.08; 95% CI= -2.34, 3.90; p=0.6220)	6.58 (SE=5.69; 95% CI=-4.57, 17.73; p=0.2595)	NR
	ACT vs MDT + amitriptyline → Pain intensity (VAS) → Depression (CES-DC) *at 7 months*	1.63 (SE=1.20; 95% CI=-0.72, 3.98; p=0.1926)	-0.13 (SE=1.67; 95% CI=-3.40, 3.14; p=0.9379)	7.42 (SE=8.74, 95% CI=-9.71, 24.55; p=0.4082)	-0.22 (SE=2.58; 90% CI=-12.16, 0.33; 95% CI=-5.28, 4.84; p=0.9332)	7.20 (SE=8.05; 95% CI=-8.58, 22.98; p=0.3835)	NR

If 95% confidence intervals not stated in original study, or only 90% confidence interval provided, then they were calculated using the standard error (indicated in red). Abbreviations: ACT, Acceptance and Commitment Therapy; ARCS, Adult Responses to Children's Symptoms; CBT, Cognitive Behavioral Therapy; CDI, Children's Depression Inventory; CES-DC, Centre for Epidemiological Studies Depression Scale for Children; CI, Confidence Interval; CSI, Children's Somatization Inventory; FDI, Functional Disability Inventory; FPS-R, Faces Pain Scale – Revised; GSRS-IBS, Gastrointestinal Symptom Rating Scale – Irritable Bowel Syndrome; IBS-BRQ, IBS-specific Behavioural Response Questionnaire; MDT, Multidisciplinary Treatment; NR, Not Reported; PBQ, PAIRS, Pain and Impairment Relationship Scale; PBQ, Pain Beliefs Questionnaire; PCQ, Pain Coping Questionnaire; PII, Pain Interference Index; PRI, Pain Response Inventory; PRS, Pain Reactivity Scale; PSS-10, Perceived Stress Scale – 10; SES, Self-Efficacy Scale; SE, Standard error; SLCBT, Social Learning and Cognitive Behavioral Treatment; TSK, Tampa Scale of Kinesiophobia; VAS, Visual Analogue Scale

eReferences

Absoud, M, Brex, P, Ciccarelli, O, Diribe, O, Giovannoni, G, Hellier, J, Howe, R, Holland, R, Kelly, J, McCrone, P, et al. (2017) A multicentre randomiSed controlled TRial of IntraVEnous immunoglobulin compared with standard therapy for the treatment of transverse myelitis in adults and children (STRIVE). *Health Technology Assessment (Winchester, England), 21*(31), 1-50. doi:10.3310/hta21310

Allantaz, F, Stichweh, D & Pascual, V (2007) Interleukin-1 as a therapeutic target in systemic-onset juvenile idiopathic arthritis. *Future Rheumatology, 2*(3), 305-312. doi:10.2217/17460816.2.3.305

Allen, KD & Shriver, MD (1998) Role of parent-mediated pain behavior management strategies in biofeedback treatment of childhood migraines. *Behavior Therapy, 29*(3), 477-490. doi:10.1016/S0005-7894(98)80044-0

Allison, DJ, Thomas, A, Beaudry, K & Ditor, DS (2016) Targeting inflammation as a treatment modality for neuropathic pain in spinal cord injury: A randomized clinical trial. *Journal of Neuroinflammation, 13*(152). doi:10.1186/s12974-016-0625-4

Altieri, F & Cassetta, M (2020) The impact of tooth-borne vs computer-guided bone-borne rapid maxillary expansion on pain and oral health–related quality of life: A parallel cohort study. *American Journal of Orthodontics and Dentofacial Orthopedics, 158*(5), e83-e90.

Anand, P, Shenoy, R, Palmer, JE, Baines, AJ, Lai, RY, Robertson, J, Bird, N, Ostenfeld, T & Chizh, BA (2011) Clinical trial of the p38 MAP kinase inhibitor dilmapimod in neuropathic pain following nerve injury. *European Journal of Pain, 15*(10), 1040-1048. doi:10.1016/j.ejpain.2011.04.005

Angst, MS, Phillips, NG, Drover, DR, Tingle, M, Ray, A, Swan, GE, Lazzeroni, LC & Clark, JD (2012) Pain sensitivity and opioid analgesia: a pharmacogenomic twin study. *Pain, 153*(7), 1397-1409. doi:10.1016/j.pain.2012.02.022

Atlas, LY, Wielgosz, J, Whittington, RA & Wager, TD (2014) Specifying the non-specific factors underlying opioid analgesia: expectancy, attention, and affect. *Psychopharmacology, 231*(5), 813-823. doi:10.1007/s00213-013-3296-1

Baccini, M, Mattei, A & Mealli, F (2017) Bayesian inference for causal mechanisms with application to a randomized study for postoperative pain control. *Biostatistics, 18*(4), 605-617. doi:10.1093/biostatistics/kxx010

Baildam, E, Ruperto, N, Brunner, H, Zuber, Z, Keane, C, Harari, O, Kenwright, A, Cuttica, RJ, Keltsev, V, Xavier, R, et al. (2013) Efficacy and safety of tocilizumab in polyarticular juvenile idiopathic arthritis: Cherish results at week 40. *Rheumatology (United Kingdom), 52*, i35. doi:10.1093/rheumatology/ket200

Barber, J & Mayer, D (1977) Evaluation of the efficacy and neural mechanism of a hypnotic analgesia procedure in experimental and clinical dental pain. *Pain, 4*(1), 41-48. doi:10.1016/0304-3959(77)90085-9

Barlow, KM, Dewey, D, Urban, KJ, Seeger, T, Kirton, A, MacMaster, F, Brooks, B, Mikrogianakis, A, Crawford, S, Netel-Aguirre, A, et al. (2014) Playgame: A randomized, double blind, placebo controlled trial of melatonin for the treatment of post concussion syndrome in youth. *Journal of Neurotrauma, 31 (12)*, A59-A60. doi:10.1089/neu.2014.9935.abstracts

Beal, SJ, Kashikar-Zuck, S, King, C, Black, W, Barnes, J & Noll, JG (2020) Heightened risk of pain in young adult women with a history of childhood maltreatment: A prospective longitudinal study. *Pain, 161*(1), 156-165. doi:http://dx.doi.org/10.1097/j.pain.0000000000001706

Bentley, MW, Stas, JM, Johnson, JM, Viet, BC & Garrett, N (2005) Effects of preincisional ketamine treatment on natural killer cell activity and postoperative pain management after oral maxillofacial surgery. *AANA journal, 73*(6), 427-436.

Blackwell, LS, Romero, SL, Marciel, KK, Romero, CV & Quittner, AL (2012) Pain in adolescents and young adults with CF: Location, severity, and predictors. *Pediatric Pulmonology, 47*, 430. doi:10.1002/ppul.22682

Bonnert, M, Bjureberg, J, Lalouni, M, Hedman, E, Serlachius, E & Ljotsson, B (2017) Reducing avoidant behaviour improves gastrointestinal symptoms in adolescents with irritable bowel syndrome: A mediation analysis of exposure-based cognitive behaviour therapy. *Journal of Pediatric Gastroenterology and Nutrition, 64 (Supplement 1)*, 132-133. doi:10.1097/01.mpg.0000516381.25680.b4

Bonnert, M, Olén, O, Bjureberg, J, Lalouni, M, Hedman-Lagerlöf, E, Serlachius, E & Ljótsson, B (2018) The role of avoidance behavior in the treatment of adolescents with irritable bowel syndrome: a mediation analysis. *Behav Res Ther, 105*, 27-35.

Bowers, H & Wroe, A (2016) Beliefs about emotions mediate the relationship between emotional suppression and quality of life in irritable bowel syndrome. *Journal of Mental Health, 25*(2), 154-158. doi:10.3109/09638237.2015.1101414

Brennan, PA, Gardiner, GT & McHugh, J (1991) A double blind clinical trial to assess the value of aprotinin in third molar surgery. *British Journal of Oral & Maxillofacial Surgery, 29*(3), 176-179. doi:10.1016/0266-4356(91)90033-2

Brown, EA, De Young, A, Kimble, R & Kenardy, J (2019) Impact of Parental Acute Psychological Distress on Young Child Pain-Related Behavior Through Differences in Parenting Behavior During Pediatric Burn Wound Care. *Journal of Clinical Psychology in Medical Settings, 04*, 04. doi:10.1007/s10880-018-9596-1

Brown, RT, Shaftman, SR, Tilley, BC, Anthony, KK, Kral, MC, Maxson, B, Mee, L, Bonner, MJ, Vogler, LB, Schanberg, LE, et al. (2012) The health education for lupus study: a randomized controlled cognitive-behavioral intervention targeting psychosocial adjustment and quality of life in adolescent females with systemic lupus erythematosus. *American Journal of the Medical Sciences, 344*(4), 274-282. doi:10.1097/MAJ.0b013e3182449be9

Brunner, HI, Ruperto, N, Quartier, P, Constantin, T, Wulffraat, N, Horneff, G, Brik, R, McCann, L, Ozdogan, H, Rutkowska-Sak, L, et al. (2012) Efficacy and safety of canakinumab in patients with active systemic juvenile idiopathic arthritis and fever: Results from two pivotal phase 3 trials. *Arthritis and Rheumatism, 64*, S326-S327. doi:10.1002/art.37735

Bryskin, RB, Londergan, B, Wheatley, R, Heng, R, Lewis, M, Barraza, M, Mercer, E & Ye, G (2015) Transversus Abdominis Plane Block Versus Caudal Epidural for Lower Abdominal Surgery in Children: A Double-Blinded Randomized Controlled Trial. *Anesthesia & Analgesia, 121*(2), 471-478. doi:10.1213/ANE.0000000000000779

Buenaver, LF, Quartana, PJ, Grace, EG, Sarlani, E, Simango, M, Edwards, RR, Haythornthwaite, JA & Smith, MT (2012) Evidence for indirect effects of pain catastrophizing on clinical pain among myofascial temporomandibular disorder participants: the mediating role of sleep disturbance. *Pain, 153*(6), 1159-1166. doi:10.1016/j.pain.2012.01.023

Caixeta, JAS, Sampaio, JCS, da Costa, PSS & Avelino, MAG (2020) Analgesia for adenotonsillectomy in children: a comparison between peritonsillar infiltration of tramadol, ketamine, and placebo. *European Archives of Oto-Rhino-Laryngology, 277*(6), 1815-1822. doi:https://dx.doi.org/10.1007/s00405-020-05878-z

Cakar Turhan, KS, Salviz, EA, Beton, S, Timuroglu, ST, Catav, S & Ozatamer, O (2015) Peritonsillar infiltration with levobupivacaine for posttonsillectomy pain relief: does concentration have any effect? A double-blind randomized controlled clinical study. *European Review for Medical & Pharmacological Sciences, 19*(7), 1276-1284.

Cappucci, S & Simons, LE (2015) Anxiety sensitivity and fear of pain in paediatric headache patients. *Eur J Pain, 19*(2), 246-252. doi:10.1002/ejp.542

Castien, R, Blankenstein, A, van der Windt, D, Heymans, MW & Dekker, J (2013) The working mechanism of manual therapy in participants with chronic tension-type headache. *Journal of Orthopaedic & Sports Physical Therapy, 43*(10), 693-699. doi:10.2519/jospt.2013.4868

Chan, SF, Connelly, M & Wallace, DP (2017) The Relationship Between Pain Characteristics, Peer Difficulties, and Emotional Functioning Among Adolescents Seeking Treatment for Chronic Pain: A Test of Mediational Models. *Journal of Pediatric Psychology, 42*(9), 941-951. doi:10.1093/jpepsy/jsx074

Chou, PL & Lin, CC (2011) A pain education programme to improve patient satisfaction with cancer pain management: a randomised control trial. *Journal of Clinical Nursing, 20*(13-14), 1858-1869. doi:10.1111/j.1365-2702.2011.03740.x

Christidis, N, Omrani, S, Fredriksson, L, Gjelset, M, Louca, S, Hedenberg-Magnusson, B & Ernberg, M (2015) Repeated tender point injections of granisetron alleviate chronic myofascial pain--a randomized, controlled,

double-blinded trial. *Journal of Headache & Pain, 16*, 104. doi:10.1186/s10194-015-0588-3
Clementi, MA, Chang, Y-H, Gambhir, R, Lebel, A & Logan, DE (2020) The Impact of Sleep on Disability and School Functioning: Results From a Tertiary Pediatric Headache Center. *Journal of Child Neurology, 35*(3), 221-227.
Conti, Y, Vatine, JJ, Levy, S, Levin Meltz, Y, Hamdan, S & Elkana, O (2020) Pain Catastrophizing Mediates the Association Between Mindfulness and Psychological Distress in Chronic Pain Syndrome. *Pain Practice*.
Corinaldesi, R, Stanghellini, V, Cremon, C, Gargano, L, Cogliandro, RF, De Giorgio, R, Bartesaghi, G, Canovi, B & Barbara, G (2009) Effect of mesalazine on mucosal immune biomarkers in irritable bowel syndrome: a randomized controlled proof-of-concept study. *Alimentary Pharmacology & Therapeutics, 30*(3), 245-252. doi:10.1111/j.1365-2036.2009.04041.x
Cunningham, NR, Kalomiris, A, Peugh, J, Farrell, M, Pentiuk, S, Mallon, D, Le, C, Moorman, E, Fussner, L & Dutta, RA (2020) Cognitive Behavior Therapy Tailored to Anxiety Symptoms Improves Pediatric Functional Abdominal Pain Outcomes: A Randomized Clinical Trial. *The Journal of Pediatrics*.
Dekker, C, Goossens, ME, Bastiaenen, CH & Verbunt, JA (2016) Study protocol for a multicentre randomized controlled trial on effectiveness of an outpatient multimodal rehabilitation program for adolescents with chronic musculoskeletal pain (2B Active). *BMC Musculoskeletal Disorders, 17*, 317. doi:10.1186/s12891-016-1178-5
DiVasta, AD, Feldman, HA, Gallagher, JS, Laufer, MR, Hornstein, MD & Gordon, CM (2015) The effect of hormonal add-back therapy in adolescents treated with a gonadotropin releasing hormone (GNRH) agonist for endometriosis: A randomized trial. *Journal of Adolescent Health, 56*(2), S24. doi:10.1016/j.jadohealth.2014.10.049
Du, SX, Jia, YR, Ren, SQ, Gong, XJ, Tang, H, Wan-Shui, W & Li-Ming, S (2018) The protective effects of Bacillus licheniformis preparation on gastrointestinal disorders and inflammation induced by radiotherapy in pediatric patients with central nervous system tumor. *Advances in Medical Sciences, 63*(1), 134-139. doi:10.1016/j.advms.2017.09.005
Dura-Ferrandis, E, Ferrando-Garcia, M, Galdon-Garrido, MJ & Andreu-Vaillo, Y (2017) Confirming the mechanisms behind cognitive-behavioural therapy effectiveness in chronic pain using structural equation modeling in a sample of patients with temporomandibular disorders. *Clinical Psychology & Psychotherapy, 24*(6), 1377-1383. doi:10.1002/cpp.2114
Essner, BS (2013) *The direct and indirect impact of pain intensity, weight status, and activity involvement on social competence outcomes in children and adolescents with spina bifida.* ProQuest Dissertations Publishing. Retrieved from http://search.proquest.com/docview/1083085872/ (347)
Evans, JR, Hainsworth, KR, Cao, Y, Khan, KA, Ladwig, R, Mano, KEJ, Davies, WH & Weisman, SJ (2014, May-June) *Examining the relationship between chronic pain*

and functioning across time - Do improvements in functional disability precede reductions in pain? Paper presented at the National Conference on Pediatric Psychology, Philadelphia, PA.
Evans, S, Cousins, L, Tsao, JC, Sternlieb, B & Zeltzer, LK (2011) Protocol for a randomized controlled study of Iyengar yoga for youth with irritable bowel syndrome. *Trials, 12*, 15. doi:10.1186/1745-6215-12-15
Evans, S, Djilas, V, Seidman, LC, Zeltzer, LK & Tsao, JCI (2017) Sleep Quality, Affect, Pain, and Disability in Children With Chronic Pain: Is Affect a Mediator or Moderator? *Journal of Pain, 18*(9), 1087-1095. doi:10.1016/j.jpain.2017.04.007
Evans, S, Payne, LA, Seidman, L, Lung, K, Zeltzer, L & Tsao, JC (2016) Maternal Anxiety and Children's Laboratory Pain: The Mediating Role of Solicitousness. *Children, 3*(2), 20. doi:10.3390/children3020010
Evans, S, Shipton, EA & Keenan, T (2006) The relationship between maternal chronic pain and child adjustment: the role of parenting as a mediator. *Journal of Pain, 7*(4), 236-243. doi:10.1016/j.jpain.2005.10.010
Fales, JL, Murphy, LK, Rights, JD & Palermo, TM (2020) Daily peer victimization experiences of adolescents with and without chronic pain: associations with mood, sleep, pain, and activity limitations. *The Journal of Pain, 21*(1-2), 97-107.
Ferrari, MD, Farkkila, M, Reuter, U, Pilgrim, A, Davis, C, Krauss, M, Diener, HC & European, COLI (2010) Acute treatment of migraine with the selective 5-HT1F receptor agonist lasmiditan--a randomised proof-of-concept trial. *Cephalalgia, 30*(10), 1170-1178. doi:10.1177/0333102410375512
Field, JJ, Kassim, A, Brandow, A, Embury, SH, Matsui, N, Wilkerson, K, Bryant, V, Zhang, L, Simpson, P & DeBaun, MR (2020) Phase 2 trial of montelukast for prevention of pain in sickle cell disease. *Blood Advances, 4*(6), 1159-1165. doi:https://dx.doi.org/10.1182/bloodadvances.2019001165
Finch, PM, Knudsen, L & Drummond, PD (2009) Reduction of allodynia in patients with complex regional pain syndrome: A double-blind placebo-controlled trial of topical ketamine. *Pain, 146*(1-2), 18-25. doi:10.1016/j.pain.2009.05.017
Fiorelli, A, Vicidomini, G, Laperuta, P, Busiello, L, Perrone, A, Napolitano, F, Messina, G & Santini, M (2010) Pre-emptive local analgesia in video-assisted thoracic surgery sympathectomy. *European Journal of Cardio-Thoracic Surgery, 37*(3), 588-593. doi:10.1016/j.ejcts.2009.07.040
Fisher, E, Caes, L, Clinch, J, Tobias, JH & Eccleston, C (2016) Anxiety at 13 and its effect on pain, pain-related anxiety, and pain-related disability at 17: An ALSPAC cohort longitudinal analysis. *Psychology Health & Medicine, 21*(1), 1-9. doi:10.1080/13548506.2015.1051062
Forsythe, LP, Thorn, B, Day, M & Shelby, G (2011) Race and sex differences in primary appraisals, catastrophizing, and experimental pain outcomes. *Journal of Pain, 12*(5), 563-572. doi:10.1016/j.jpain.2010.11.003
Foxen-Craft, EC (2017) *Exercise-induced hypoalgesia: Brief submaximal exercise, acute cold pressor pain, and psychological mediators in young adults.* ProQuest Dissertations Publishing. Retrieved from http://search.proquest.com/docview/1825633748/

Garland, EL, Gaylord, SA, Palsson, O, Faurot, K, Douglas Mann, J & Whitehead, WE (2012) Therapeutic mechanisms of a mindfulness-based treatment for IBS: effects on visceral sensitivity, catastrophizing, and affective processing of pain sensations. *Journal of Behavioral Medicine, 35*(6), 591-602. doi:10.1007/s10865-011-9391-z

Gaultney, WM (2020) An exploration of dispositional mindfulness and the mechanisms of pain processing in children. *Dissertation Abstracts International: Section B: The Sciences and Engineering, 81*(2-B).

Ghorbani, Z, Rafiee, P, Fotouhi, A, Haghighi, S, Magham, RR, Ahmadi, ZS, Djalali, M, Zareei, M, Jahromi, SR & Shahemi, S (2020) The effects of vitamin D supplementation on interictal serum levels of calcitonin gene-related peptide (CGRP) in episodic migraine patients: post hoc analysis of a randomized double-blind placebo-controlled trial. *The Journal of Headache and Pain, 21*(1), 1-13.

Gillis, ME (2002) *The effects of written emotional disclosure on adjustment in fibromyalgia syndrome.* ProQuest Dissertations Publishing. Retrieved from http://search.proquest.com/docview/287863044/

Glenn, JJ, Michel, BD, Franklin, JC, Hooley, JM & Nock, MK (2014) Pain analgesia among adolescent self-injurers. *Psychiatry Research, 220*(3), 921-926. doi:10.1016/j.psychres.2014.08.016

Gomez-Mancilla, B, Cutler, NR, Leibowitz, MT, Spierings, EL, Klapper, JA, Diamond, S, Goldstein, J, Smith, T, Couch, JR, Fleishaker, J, et al. (2001) Safety and efficacy of PNU-142633, a selective 5-HT1D agonist, in patients with acute migraine. *Cephalalgia, 21*(7), 727-732. doi:10.1111/j.1468-2982.2001.00208.x

Gottschlich, MM, Mayes, T, Khoury, J, McCall, J, Simakajornboon, N & Kagan, RJ (2011) The effect of ketamine administration on nocturnal sleep architecture. *Journal of Burn Care & Research, 32*(5), 535-540. doi:10.1097/BCR.0b013e31822ac7d1

Grinsvall, C, Tornblom, H, Tack, J, Van Oudenhove, L & Simren, M (2015) The effect of GI-specific anxiety and abuse on visceral sensitivity in irritable bowel syndrome is mediated through somatization. *United European Gastroenterology Journal, 3*(5), A637-A638. doi:10.1177/2050640615601623

Harel, Z, Riggs, S, Vaz, R, Flanagan, P & Harel, D (2004) The use of the leukotriene receptor antagonist montelukast (Singulair) in the management of dysmenorrhea in adolescents. *Journal of Pediatric & Adolescent Gynecology, 17*(3), 183-186. doi:10.1016/j.jpag.2004.03.037

Harper, FW, Penner, LA, Peterson, A, Albrecht, TL & Taub, J (2012) Children's positive dispositional attributes, parents' empathic responses, and children's responses to painful pediatric oncology treatment procedures. *Journal of Psychosocial Oncology, 30*(5), 593-613. doi:10.1080/07347332.2012.703771

Hashish, I, Hai, HK, Harvey, W, Feinmann, C & Harris, M (1988) Reduction of postoperative pain and swelling by ultrasound treatment: a placebo effect. *Pain, 33*(3), 303-311. doi:10.1016/0304-3959(88)90289-8

He, HG, Zhu, L, Chan, WC, Xiao, C, Klainin-Yobas, P, Wang, W, Cheng, KF & Luo, N (2015) A randomized controlled trial of the effectiveness of an educational intervention on outcomes of parents and their children undergoing inpatient elective surgery: study protocol. *Journal of Advanced Nursing, 71*(3), 665-675. doi:10.1111/jan.12521

Hechler, T, Dobe, M, Damschen, U, Blankenburg, M, Schroeder, S, Kosfelder, J & Zernikow, B (2010) The pain provocation technique for adolescents with chronic pain: preliminary evidence for its effectiveness. *Pain Medicine, 11*(6), 897-910. doi:10.1111/j.1526-4637.2010.00839.x

Heeney, M, Hoppe, C, Abboud, M, Inusa, B, Kanter, J, Ogutu, B, Brown, P, Heath, L, Jakubowski, J, Zhou, C, et al. (2016a, June) *Determining effects of platelet inhibition on vaso-occlusive events (DOVE) trial: A double-blind, placebo-controlled, study of prasugrel in paediatric patients with sickle cell anaemia.* Paper presented at the 21st Congress of the European Hematology Association, Denmark.

Heeney, MM, Abboud, MR, Amilon, C, Andersson, M, Githanga, J, Inusa, B, Kanter, J, Leonsson-Zachrisson, M, Michelson, AD & Berggren, AR (2018) Ticagrelor versus placebo for the reduction of vasoocclusive crises in pediatric sickle cell disease: Design of a randomized, double-blind, parallel-group, multicenter phase 3 study (HESTIA3). *HemaSphere, 2 (Supplement 2)*, 669-670. doi:10.1097/HS9.0000000000000060

Heeney, MM, Hoppe, CC, Abboud, MR, Inusa, B, Kanter, J, Ogutu, B, Brown, PB, Heath, LE, Jakubowski, JA, Zhou, C, et al. (2016b) A Multinational Trial of Prasugrel for Sickle Cell Vaso-Occlusive Events. *New England Journal of Medicine, 374*(7), 625-635. doi:10.1056/NEJMoa1512021

Herroeder, S, Pecher, S, Schonherr, ME, Kaulitz, G, Hahnenkamp, K, Friess, H, Bottiger, BW, Bauer, H, Dijkgraaf, MG, Durieux, ME, et al. (2007) Systemic lidocaine shortens length of hospital stay after colorectal surgery: a double-blinded, randomized, placebo-controlled trial. *Annals of Surgery, 246*(2), 192-200. doi:10.1097/SLA.0b013e31805dac11

Hildenbrand, AK, Kassam-Adams, N, Barakat, LP, Kohser, KL, Ciesla, JA, Delahanty, DL, Fein, JA, Ragsdale, LB & Marsac, ML (2020) Posttraumatic stress in children after injury: The role of acute pain and opioid medication use. *Pediatric emergency care.*

Hillgrove-Stuart, J, Pillai Riddell, R, Horton, R & Greenberg, S (2013) Toy-mediated distraction: clarifying the role of agent of distraction and preneedle distress in toddlers. *Pain Research & Management, 18*(4), 197-202. doi:10.1155/2013/392125

Hilt, LM (2009) *A biopsychosocial approach to rumination in young adolescent girls.* ProQuest Dissertations Publishing. Retrieved from http://search.proquest.com/docview/305040452/

Hind, D, Parkin, J, Whitworth, V, Rex, S, Young, T, Hampson, L, Sheehan, J, Maguire, C, Cantrill, H, Scott, E, et al. (2017) Aquatic therapy for children with Duchenne muscular dystrophy: a pilot feasibility randomised controlled

trial and mixed-methods process evaluation. *Health Technology Assessment (Winchester, England), 21*(27), 1-120. doi:10.3310/hta21270

Hooke, MC, Rodgers, C, Taylor, O, Koerner, KM, Mitby, P, Moore, I, Scheurer, ME, Hockenberry, MJ & Pan, W (2018) Physical Activity, the Childhood Cancer Symptom Cluster-Leukemia, and Cognitive Function: A Longitudinal Mediation Analysis. *Cancer Nursing, 41*(6), 434-440. doi:10.1097/NCC.0000000000000634

Hoyeraal, HM, Froland, SS & Salvesen, CF (1978) No effect of transfer factor in juvenile rheumatoid arthritis by double blind trial. *Annals of the Rheumatic Diseases, 37*(2), 175-179. doi:10.1136/ard.37.2.175

Ingelmo, PM, Bendall, EJ, Frawley, G, Locatelli, BG, Milan, B, Lodetti, D & Fumagalli, R (2007) Bupivacaine caudal epidural anesthesia: assessing the effect of general anesthetic technique on block onset. *Paediatric Anaesthesia, 17*(3), 255-262. doi:10.1111/j.1460-9592.2006.02090.x

Janssens, KAM, Oldehinkel, AJ, Bonvanie, IJ & Rosmalen, JGM (2014) An inactive lifestyle and low physical fitness are associated with functional somatic symptoms in adolescents. The TRAILS study. *Journal of Psychosomatic Research, 76*(6), 454-457. doi:10.1016/j.jpsychores.2014.03.008

Jones, J, Gordish-Dressman, H, Pestieau, S & Reddy, SK (2018, April) *Dexmedetomidine as a non-opiate adjunct to multimodal perioperative pain control in pediatric patients undergoing craniofacial reconstructive surgery: A retrospective study.* Paper presented at the George Washington Research Days, George Washington University.

Jonsbu, E, Dammen, T, Morken, G, Moum, T & Martinsen, EW (2011) Short-term cognitive behavioral therapy for non-cardiac chest pain and benign palpitations: a randomized controlled trial. *Journal of Psychosomatic Research, 70*(2), 117-123. doi:10.1016/j.jpsychores.2010.09.013

Junghans-Rutelonis, AN, Craner, JR, Ale, CM, Harbeck-Weber, C, Fischer, PR & Weiss, KE (2018) Youth with Chronic Pain and Postural Orthostatic Tachycardia Syndrome (POTS): Treatment Mediators of Improvement in Functional Disability. *Journal of Clinical Psychology in Medical Settings, 25*(4), 471-484. doi:10.1007/s10880-018-9558-7

Kashikar-Zuck, S, Sil, S, Lynch-Jordan, AM, Ting, TV, Peugh, J, Schikler, KN, Hashkes, PJ, Arnold, LM, Passo, M & Richards-Mauze, MM (2013) Changes in pain coping, catastrophizing, and coping efficacy after cognitive-behavioral therapy in children and adolescents with juvenile fibromyalgia. *J Pain, 14*(5), 492-501.

Khayat, S, Fanaei, H, Kheirkhah, M, Moghadam, ZB, Kasaeian, A & Javadimehr, M (2015) Curcumin attenuates severity of premenstrual syndrome symptoms: A randomized, double-blind, placebo-controlled trial. *Complementary Therapies in Medicine, 23*(3), 318-324. doi:10.1016/j.ctim.2015.04.001

Kilkens, TO, Honig, A, Fekkes, D & Brummer, RJ (2005) The effects of an acute serotonergic challenge on brain-gut responses in irritable bowel syndrome

patients and controls. *Alimentary Pharmacology & Therapeutics, 22*(9), 865-874. doi:10.1111/j.1365-2036.2005.02660.x
Kobayashi, I, Tozawa, Y, Ueki, M, Takezaki, S, Watanabe, S, Iwafuchi, H, Yamada, M, Kuwana, M & Ariga, T (2017) Tacrolimus in combination with methotrexate and corticosteroid for the treatment of child-onset anti-signal recognition particle antibody-positive necrotizing myopathy. *Scandinavian Journal of Rheumatology, 46*(5), 409-410. doi:10.1080/03009742.2016.1241297
Kościelniak-Merak, B, Batko, I, Kobylarz, K, Sztefko, K & Tomasik, PJ (2020) Intravenous, perioperatively administered lidocaine regulates serum pain modulators' concentrations in children undergoing spinal surgery. *Pain Medicine, 21*(7), 1464-1473.
Lai, HH, Chiu, CH, Kong, MS, Chang, CJ & Chen, CC (2019) Probiotic Lactobacillus casei: effective for Managing Childhood Diarrhea by Altering Gut Microbiota and Attenuating Fecal Inflammatory Markers. *Nutrients, 11*(5). doi:10.3390/nu11051150
Lalouni, M, Hesser, H, Bonnert, M, Hedman-Lagerlöf, E, Serlachius, E, Olén, O & Ljótsson, B (2020) Breaking the vicious circle of fear and avoidance in children with abdominal pain: A mediation analysis. *J Psychosom Res, 140*, 110287.
Langer, S, Romano, J, Mancl, L & Levy, RL (2013) Parental catastrophizing mediates the association between child pain behavior and parental solicitousness. *Psychosomatic Medicine, 75 (3)*, A-46. doi:10.1097/01.psy.0000429452.16135.1b
Langer, SL, Romano, JM, Mancl, L & Levy, RL (2014) Parental Catastrophizing Partially Mediates the Association between Parent-Reported Child Pain Behavior and Parental Protective Responses. *Pain Research and Treatment, 2014*, 751097. doi:10.1155/2014/751097
Lee, A, Moulton, D, Acra, S, Walker, L, McKernan, L & Russell, A (2020) Clinical Hypnosis in Pediatric Crohn's Disease: A Randomized Controlled Trial. *Gastroenterology, 158 (3 Supplement)*, S100-S101. doi:http://dx.doi.org/10.1053/j.gastro.2019.11.239
Leeuw, M, Goossens, ME, van Breukelen, GJ, de Jong, JR, Heuts, PH, Smeets, RJ, Koke, AJ & Vlaeyen, JW (2008) Exposure in vivo versus operant graded activity in chronic low back pain patients: results of a randomized controlled trial. *Pain, 138*(1), 192-207. doi:10.1016/j.pain.2007.12.009
Levy, R, Langer, S, Labus, J, DuPen, M, Ballard, S & Romano, J (2012) Changes in pain cognitions mediate effects of cognitive-behavioral treatment on reductions in symptoms and disability in children with unexplained abdominal pain. *American Journal of Gastroenterology, 107*, S782. doi:10.1038/ajg.2012.278
Levy, RL, Langer, SL, Romano, JM, Labus, J, Walker, LS, Murphy, TB, Van Tilburg, M, Feld, LD, Christie, DL & Whitehead, WE (2014) Cognitive mediators of treatment outcomes in pediatric functional abdominal pain. *Clin J Pain, 30*(12), 1033.

Lewis, HA & Kliewer, W (1996) Hope, coping, and adjustment among children with sickle cell disease: Tests of mediator and moderator models. *Journal of Pediatric Psychology, 21*(1), 25-41. doi:10.1093/jpepsy/21.1.25

Li, Q, Li, L, Bi, L, Xiao, C, Lin, Z, Cao, S, Liao, Z & Gu, J (2016) Kunxian capsules in the treatment of patients with ankylosing spondylitis: a randomized placebo-controlled clinical trial. *Trials, 17*(1), 337. doi:10.1186/s13063-016-1438-6

Lim, CS, Karlson, C, Edmond, SN, Welkom, JS, Osunkwo, I & Cohen, LL (2019) Emotion-Focused Avoidance Coping Mediates the Association Between Pain and Health-Related Quality of Life in Children With Sickle Cell Disease. *Journal of Pediatric Hematology/Oncology, 41*(3), 194-201. doi:10.1097/MPH.0000000000001429

Liossi, C, White, P, Franck, L & Hatira, P (2007) Parental pain expectancy as a mediator between child expected and experienced procedure-related pain intensity during painful medical procedures. *Clinical Journal of Pain, 23*(5), 392-399. doi:10.1097/AJP.0b013e31804ac00c

Lohsiriwat, V, Lert-akyamanee, N & Rushatamukayanunt, W (2004) Efficacy of pre-incisional bupivacaine infiltration on postoperative pain relief after appendectomy: prospective double-blind randomized trial. *World Journal of Surgery, 28*(10), 947-950. doi:10.1007/s00268-004-7471-8

Love, SC, Mara, CA, Kalomiris, AE & Cunningham, NR (2019) The Influence of Caregiver Distress and Child Anxiety in Predicting Child Somatization in Youth with Functional Abdominal Pain Disorders. *Children, 6*(12), 03. doi:https://dx.doi.org/10.3390/children6120134

Lowen, MB, Mayer, EA, Sjoberg, M, Tillisch, K, Naliboff, B, Labus, J, Lundberg, P, Strom, M, Engstrom, M & Walter, SA (2013) Effect of hypnotherapy and educational intervention on brain response to visceral stimulus in the irritable bowel syndrome. *Alimentary Pharmacology & Therapeutics, 37*(12), 1184-1197. doi:10.1111/apt.12319

Lu, P, Hsu, J, Keane, C, Fettner, S, Wang, J, Ruperto, N, Harari, O, Brunner, HI & De Benedetti, F (2013) PReS-FINAL-2159: Tocilizumab (TCZ) dosing in juvenile idiopathic arthritis (JIA): Optimising for different JIA type and body weight patients. *Pediatric Rheumatology. Conference: 20th Pediatric Rheumatology European Society, PReS Congress. Ljubljana Slovenia. Conference Publication:, 11*(SUPPL. 2). doi:10.1186/1546-0096-11-S2-P171

Luciano, JV, Guallar, JA, Aguado, J, Lopez-Del-Hoyo, Y, Olivan, B, Magallon, R, Alda, M, Serrano-Blanco, A, Gili, M & Garcia-Campayo, J (2014) Effectiveness of group acceptance and commitment therapy for fibromyalgia: a 6-month randomized controlled trial (EFFIGACT study). *Pain, 155*(4), 693-702. doi:10.1016/j.pain.2013.12.029

Lustig, JL, Ireys, HT, Sills, EM & Walsh, BB (1996) Mental health of mothers of children with juvenile rheumatoid arthritis: appraisal as a mediator. *Journal of Pediatric Psychology, 21*(5), 719-733. doi:10.1093/jpepsy/21.5.719

Maddison, R, Prapavessis, H & Clatworthy, M (2006) Modeling and rehabilitation following anterior cruciate ligament reconstruction. *Annals of Behavioral Medicine, 31*(1), 89-98. doi:10.1207/s15324796abm3101_13

Malattia, C, Ruperto, N, Pederzoli, S, Palmisani, E, Pistorio, A, Wouters, C, Dolezalova, P, Flato, B, Garay, S & Giancane, G (2020) Tocilizumab may slow radiographic progression in patients with systemic or polyarticular-course juvenile idiopathic arthritis: post hoc radiographic analysis from two randomized controlled trials. *Arthritis research & therapy, 22*(1), 1-11.

Mansell, G, Kamper, SJ & Kent, P (2013) Why and how back pain interventions work: what can we do to find out? *Best Pract Res Clin Rheumatol, 27*(5), 685-697.

McGarrigle, L, Ferreira, N, DeAmicis, L & Connolly, S (2018) Pediatric chronic pain and adjustment: The mediating role of acceptance, catastrophizing and kinesiophobia. *Pain Practice, 18 (Supplement 1)*, 103. doi:10.1111/papr.12693

Meier, PM, Zurakowski, D, Berde, CB & Sethna, NF (2009) Lumbar sympathetic blockade in children with complex regional pain syndromes: a double blind placebo-controlled crossover trial. *Anesthesiology, 111*(2), 372-380. doi:10.1097/ALN.0b013e3181aaea90

Melzack, R, Guite, S & Gonshor, A (1980a) Relief of dental pain by ice massage of the hand. *Canadian Medical Association Journal, 122*(2), 189-191.

Melzack, R, Jeans, ME, Stratford, JG & Monks, RC (1980b) Ice massage and transcutaneous electrical stimulation: comparison of treatment for low-back pain. *Pain, 9*(2), 209-217. doi:10.1016/0304-3959(80)90008-1

Merskey, H & Bogduk, N (1994) *Classification of chronic pain: descriptions of chronic pain syndromes and definitions of pain terms* (2nd ed.). Seattle: IASP Press.

Miller, M, Williams, A, Scott, E, Trost, Z & Hirsh, A (2017) Pain-related injustice perception, but not catastrophizing, partially mediates the relationship between pain intensity and pain outcomes in children with chronic pain. *Journal of Pain, 18*, S41. doi:10.1016/j.jpain.2017.02.154

Milling, LS, Reardon, JM & Carosella, GM (2006) Mediation and moderation of psychological pain treatments: response expectancies and hypnotic suggestibility. *Journal of Consulting & Clinical Psychology, 74*(2), 253-262. doi:10.1037/0022-006X.74.2.253

Milling, LS, Shores, JS, Coursen, EL, Menario, DJ & Farris, CD (2007) Response expectancies, treatment credibility, and hypnotic suggestibility: mediator and moderator effects in hypnotic and cognitive-behavioral pain interventions. *Annals of Behavioral Medicine, 33*(2), 167-178. doi:10.1007/bf02879898

Mohammed, FA, Mahdi, N, Sater, MA, Al-Ola, K & Almawi, WY (2010) The relation of C-reactive protein to vasoocclusive crisis in children with sickle cell disease. *Blood Cells Molecules & Diseases, 45*(4), 293-296. doi:10.1016/j.bcmd.2010.08.003

Moore, UJ, Seymour, RA & Rawlins, MD (1992) The efficacy of locally applied aspirin and acetaminophen in postoperative pain after third molar surgery. *Clinical Pharmacology & Therapeutics, 52*(3), 292-296. doi:10.1038/clpt.1992.144

Mulroy, SJ, Winstein, CJ, Kulig, K, Beneck, GJ, Fowler, EG, DeMuth, SK, Sullivan, KJ, Brown, DA, Lane, CJ & Physical Therapy Clinical Research, N (2011) Secondary mediation and regression analyses of the PTClinResNet database: determining causal relationships among the International Classification of Functioning, Disability and Health levels for four physical therapy intervention trials. *Physical Therapy, 91*(12), 1766-1779. doi:10.2522/ptj.20110024

Neville, A, Kopala-Sibley, DC, Soltani, S, Asmundson, GJ, Jordan, A, Carleton, RN, Yeates, KO, Schulte, F & Noel, M (2020) A longitudinal examination of the interpersonal fear avoidance model of pain: the role of intolerance of uncertainty. *Pain, 162*(1), 152-160.

Nickel, JC, Atkinson, G, Krieger, JN, Mills, IW, Pontari, M, Shoskes, DA & Crook, TJ (2012) Preliminary assessment of safety and efficacy in proof-of-concept, randomized clinical trial of tanezumab for chronic prostatitis/chronic pelvic pain syndrome. *Urology, 80*(5), 1105-1110. doi:10.1016/j.urology.2012.07.035

Niedermann, K, de Bie, RA, Kubli, R, Ciurea, A, Steurer-Stey, C, Villiger, PM & Buchi, S (2011) Effectiveness of individual resource-oriented joint protection education in people with rheumatoid arthritis. A randomized controlled trial. *Patient Education & Counseling, 82*(1), 42-48. doi:10.1016/j.pec.2010.02.014

Noel, M, Rabbitts, JA, Tai, GG & Palermo, TM (2015) Remembering pain after surgery: a longitudinal examination of the role of pain catastrophizing in children's and parents' recall. *Pain, 156*(5), 800-808. doi:10.1097/j.pain.0000000000000102

Noel, M, Vinall, J, Tomfohr-Madsen, L, Holley, AL, Wilson, AC & Palermo, TM (2018) Sleep Mediates the Association Between PTSD Symptoms and Chronic Pain in Youth. *Journal of Pain, 19*(1), 67-75. doi:10.1016/j.jpain.2017.09.002

Olbrecht, VA, Ding, L, Spruance, K, Hossain, M, Sadhasivam, S & Chidambaran, V (2018) Intravenous acetaminophen reduces length of stay via mediation of postoperative opioid consumption after posterior spinal fusion in a pediatric cohort. *Clin J Pain, 34*(7), 593-599.

Palermo, TM, Zempsky, WT, Dampier, CD, Lalloo, C, Hundert, AS, Murphy, LK, Bakshi, N & Stinson, JN (2018) iCanCope with Sickle Cell Pain: Design of a randomized controlled trial of a smartphone and web-based pain self-management program for youth with sickle cell disease. *Contemporary Clinical Trials, 74*, 88-96. doi:10.1016/j.cct.2018.10.006

Palstam, A, Larsson, A, Lofgren, M, Ernberg, M, Bjersing, J, Bileviciute-Ljungar, I, Gerdle, B, Kosek, E & Mannerkorpi, K (2016) Decrease of fear avoidance beliefs following person-centered progressive resistance exercise contributes to reduced pain disability in women with fibromyalgia: secondary exploratory analyses from a randomized controlled trial. *Arthritis Research & Therapy, 18*(1), 116. doi:10.1186/s13075-016-1007-0

Pavlova, M, Ference, J, Hancock, M & Noel, M (2017) Disentangling the Sleep-Pain Relationship in Pediatric Chronic Pain: The Mediating Role of Internalizing Mental Health Symptoms. *Pain Research & Management, 2017*, 1586921. doi:10.1155/2017/1586921

Pavlova, M, Kopala-Sibley, DC, Nania, C, Mychasiuk, R, Christensen, J, McPeak, A, Tomfohr-Madsen, L, Katz, J, Palermo, TM & Noel, M (2020) Sleep disturbance underlies the co-occurrence of trauma and pediatric chronic pain: a longitudinal examination. *Pain, 161*(4), 821-830. doi:https://dx.doi.org/10.1097/j.pain.0000000000001769

Pavlova, M & Noel, M (2018) JPP student journal club commentary: Novel parent intervention reduces vaccine injection pain in toddlers: Potential mechanisms and path forward. *Journal of Pediatric Psychology, 43*(3), 234-236. doi:10.1093/jpepsy/jsx152

Peatfield, RC, Petty, RG & Rose, FC (1983) Double blind comparison of mefenamic acid and acetaminophen (paracetamol) in migraine. *Cephalalgia, 3*(2), 129-134. doi:10.1046/j.1468-2982.1983.0302129.x

Petter, M, McGrath, PJ, Chambers, CT & Dick, BD (2014) The effects of mindful attention and state mindfulness on acute experimental pain among adolescents. *Journal of Pediatric Psychology, 39*(5), 521-531. doi:10.1093/jpepsy/jsu007

Peugh, JL, Strotman, D, McGrady, M, Rausch, J & Kashikar-Zuck, S (2017) Beyond intent to treat (ITT): A complier average causal effect (CACE) estimation primer. *Journal of School Psychology, 60*, 7-24. doi:10.1016/j.jsp.2015.12.006

Poppert Cordts, KM, Stone, AL, Beveridge, JK, Wilson, AC & Noel, M (2019) The (Parental) Whole Is Greater Than the Sum of Its Parts: A Multifactorial Model of Parent Factors in Pediatric Chronic Pain. *Journal of Pain, 20*(7), 786-795. doi:10.1016/j.jpain.2019.01.004

Posner, KL (1999) *A pharmacobehavioral intervention to reduce child cancer distress.* ProQuest Dissertations Publishing. Retrieved from http://search.proquest.com/docview/304460697/

Pringsheim, T, Magnoux, E, Dobson, CF, Hamel, E & Aube, M (2002) Melatonin as adjunctive therapy in the prophylaxis of cluster headache: a pilot study. *Headache, 42*(8), 787-792. doi:10.1046/j.1526-4610.2002.02181.x

Puzino, K, Guite, JW, Moore, M, Lewen, MO & Williamson, AA (2018) The relationship between parental responses to pain, pain catastrophizing, and adolescent sleep in adolescents with chronic pain. *Children's Health Care, 47*(3), 239-260. doi:10.1080/02739615.2017.1327358

Quispe-Cabanillas, JG, Damasceno, A, von Glehn, F, Brandao, CO, Damasceno, BP, Silveira, WD & Santos, LM (2012) Impact of electroacupuncture on quality of life for patients with Relapsing-Remitting Multiple Sclerosis under treatment with immunomodulators: a randomized study. *BMC Complementary & Alternative Medicine, 12*, 209. doi:10.1186/1472-6882-12-209

Randall, ET, Cole-Lewis, YC, Petty, CR & Jervis, KN (2020) Understanding How Perfectionism Impacts Intensive Interdisciplinary Pain Treatment Outcomes: A Nonrandomized Trial. *Journal of Pediatric Psychology*.

Reddy, SK, Jones, JJ, Gordish-Dressman, H & Pestieau, SR (2020) Dexmedetomidine as an opioid-sparing agent in pediatric craniofacial surgery. *Children, 7*(7), 68.

Reed-Knight, B, van Tilburg, MAL, Levy, RL, Langer, SL, Romano, JM, Murphy, TB, DuPen, MM & Feld, AD (2018) Maladaptive Coping and Depressive Symptoms Partially Explain the Association Between Family Stress and Pain-Related Distress in Youth With IBD. *Journal of Pediatric Psychology, 43*(1), 94-103. doi:10.1093/jpepsy/jsx082

Reid, JK (2003) *Family characteristics and caregiver psychosocial factors related to health outcomes in children with sickle cell disease: A mediational model.* ProQuest Dissertations Publishing. Retrieved from http://search.proquest.com/docview/305498714/

Reme, SE, Stahl, D, Kennedy, T, Jones, R, Darnley, S & Chalder, T (2011) Mediators of change in cognitive behaviour therapy and mebeverine for irritable bowel syndrome. *Psychological Medicine, 41*(12), 2669-2679. doi:10.1017/S0033291711000328

Riggenbach, A, Amouroux, R, Van Petegem, S, Tourniaire, B, Tonelli, A, Wiener, S, Hofer, M & Antonietti, JP (2020) Autonomy and competence satisfaction as resources for facing chronic pain disability in adolescence: a self-determination perspective. *Psychology, Health & Medicine*, 1-11.

Robinson, JP, Theodore, BR, Dansie, EJ, Wilson, HD & Turk, DC (2013) The role of fear of movement in subacute whiplash-associated disorders grades I and II. *Pain, 154*(3), 393-401. doi:10.1016/j.pain.2012.11.011

Rolli Salathe, C, Kalin, W, Zilse, S & Elfering, A (2020) Baseline musculoskeletal pain and impaired sleep related to school pressure influence the development of musculoskeletal pain in N = 107 adolescents in a 5-year longitudinal study. *European Spine Journal, 29*(3), 540-548. doi:https://dx.doi.org/10.1007/s00586-019-06211-x

Ruperte, N, Zhang, A, Morcos, PN, Huppertz, H, Minden, K, Myones, B, Onel, K, Siammopoulou, A, Silva, C, Kenwright, A, et al. (2011) Tocilizumab in patients with systemic juvenile idiopathic arthritis (sJIA): 12-week pharmacokinetic (PK) and pharmacodynamic (PD) data from the phase 3 TENDER trial. *Clinical and Experimental Rheumatology, 29*(2), 421.

Ruperto, N, Brunner, H, Quartier, P, Constantin, T, Wulffraat, N, Horneff, G, Brik, R, McCann, L, Kasapcopur, O, Rutkowska-Sak, L, et al. (2013) Efficacy and safety of canakinumab, fully human anti-interleukin-1beta antibody, in systemic juvenile idiopathic arthritis. *Annals of the Rheumatic Disease, 71*. doi:10.1136/annrheumdis-2012-eular.1180

Saxe, G, Geary, M, Bedard, K, Bosquet, M, Miller, A, Koenen, K, Stoddard, F & Moulton, S (2006) Separation anxiety as a mediator between acute morphine

administration and PTSD symptoms in injured children. *Annals of the New York Academy of Sciences, 1071*, 41-45. doi:10.1196/annals.1364.004

Schoenen, J, Jensen, RH, Lanteri-Minet, M, Lainez, MJ, Gaul, C, Goodman, AM, Caparso, A & May, A (2013) Stimulation of the sphenopalatine ganglion (SPG) for cluster headache treatment. Pathway CH-1: a randomized, sham-controlled study. *Cephalalgia, 33*(10), 816-830. doi:10.1177/0333102412473667

Schreiber, S, Vinokur, S, Shavelzon, V, Pick, CG, Zahavi, E & Shir, Y (2001) A randomized trial of fluoxetine versus amitriptyline in musculo-skeletal pain. *Israel Journal of Psychiatry & Related Sciences, 38*(2), 88-94.

Schurman, JV, Friesen, CA, Dai, H, Danda, CE, Hyman, PE & Cocjin, JT (2012) Sleep problems and functional disability in children with functional gastrointestinal disorders: an examination of the potential mediating effects of physical and emotional symptoms. *BMC Gastroenterology, 12*, 142. doi:10.1186/1471-230X-12-142

Shah, UK, Galinkin, J, Chiavacci, R & Briggs, M (2002) Tonsillectomy by means of plasma-mediated ablation: prospective, randomized, blinded comparison with monopolar electrosurgery. *Archives of Otolaryngology -- Head & Neck Surgery, 128*(6), 672-676. doi:10.1001/archotol.128.6.672

Shi, GX, Liu, CZ, Zhu, J, Guan, LP, Wang, DJ & Wu, MM (2011) Effects of acupuncture at Sanyinjiao (SP6) on prostaglandin levels in primary dysmenorrhea patients. *Clinical Journal of Pain, 27*(3), 258-261. doi:10.1097/AJP.0b013e3181fb27ae

Shi, Y, Wang, P, Hu, X & Ye, Z (2015) Evaluation of the Etoricoxib-Mediated Pain-Relieving Effect in Patients Undergoing Lumbar Fusion Procedures for Degenerative Lumbar Scoliosis: A Prospective Randomized, Double-Blind Controlled Study. *Cell Biochemistry & Biophysics, 71*(3), 1313-1318. doi:10.1007/s12013-014-0350-5

Sieberg, CB, Williams, S & Simons, LE (2011) Do parent protective responses mediate the relation between parent distress and child functional disability among children with chronic pain? *Journal of Pediatric Psychology, 36*(9), 1043-1051. doi:10.1093/jpepsy/jsr043

Simister, HD, Tkachuk, GA, Shay, BL, Vincent, N, Pear, JJ & Skrabek, RQ (2018) Randomized Controlled Trial of Online Acceptance and Commitment Therapy for Fibromyalgia. *Journal of pain, 19*(7), 741-753. doi:10.1016/j.jpain.2018.02.004

Slaman, J, van den Berg-Emons, HJ, van Meeteren, J, Twisk, J, van Markus, F, Stam, HJ, van der Slot, WM & Roebroeck, ME (2015) A lifestyle intervention improves fatigue, mental health and social support among adolescents and young adults with cerebral palsy: focus on mediating effects. *Clinical Rehabilitation, 29*(7), 717-727. doi:10.1177/0269215514555136

Spinhoven, P, Ter Kuile, M, Kole-Snijders, AM, Hutten Mansfeld, M, Den Ouden, DJ & Vlaeyen, JW (2004) Catastrophizing and internal pain control as mediators

of outcome in the multidisciplinary treatment of chronic low back pain. *European Journal of Pain, 8*(3), 211-219. doi:10.1016/j.ejpain.2003.08.003

Stratelak, PA, White, W & Wenzel, D (1996) The effect of glycopyrrolate premedication on postoperative sore throat. *AANA Journal, 64*(6), 545-548.

Taheri, S, Campbell, C, Mazza, E & Devries, M (2016) Randomized controlled trial of IGG-based food elimination for treatment of primary headache in children-interim analysis. *Cephalalgia, 36 (Supplement 1)*, 89. doi:10.1177/0333102416670318

Tarnowski, KJ, McGrath, ML, Calhoun, MB & Drabman, RS (1987) Pediatric burn injury: self- versus therapist-mediated debridement. *Journal of Pediatric Psychology, 12*(4), 567-579. doi:10.1093/jpepsy/12.4.567

ter Kuile, MM, Spinhoven, P, Linssen, AC & van Houwelingen, HC (1996) Cognitive coping and appraisal processes in the treatment of chronic headaches. *Pain, 64*(2), 257-264. doi:10.1016/0304-3959(95)00135-2

Thieme, K, Turk, DC, Gracely, RH, Maixner, W & Flor, H (2015) The relationship among psychological and psychophysiological characteristics of fibromyalgia patients. *Journal of Pain, 16*(2), 186-196. doi:10.1016/j.jpain.2014.11.009

Tran, S (2015) *Longitudinal associations between anxiety, pain catastrophizing, and treatment outcomes in complex pediatric chronic pain.* ProQuest Dissertations Publishing. Retrieved from http://search.proquest.com/docview/1615378112/

Tran, ST, Jastrowski Mano, KE, Hainsworth, KR, Medrano, GR, Anderson Khan, K, Weisman, SJ & Davies, WH (2015) Distinct Influences of Anxiety and Pain Catastrophizing on Functional Outcomes in Children and Adolescents With Chronic Pain. *Journal of Pediatric Psychology, 40*(8), 744-755. doi:10.1093/jpepsy/jsv029

Tremblay, I & Sullivan, MJ (2010) Attachment and pain outcomes in adolescents: the mediating role of pain catastrophizing and anxiety. *Journal of Pain, 11*(2), 160-171. doi:10.1016/j.jpain.2009.06.015

Troullos, ES, Hargreaves, KM, Butler, DP & Dionne, RA (1990) Comparison of nonsteroidal anti-inflammatory drugs, ibuprofen and flurbiprofen, with methylprednisolone and placebo for acute pain, swelling, and trismus. *Journal of Oral and Maxillofacial Surgery, 48*(9), 945-952. doi:10.1016/0278-2391(90)90007-O

Tsao, JC, Lu, Q, Kim, SC & Zeltzer, LK (2006a) Relationships among anxious symptomatology, anxiety sensitivity and laboratory pain responsivity in children. *Cognitive Behaviour Therapy, 35*(4), 207-215. doi:10.1080/16506070600898272

Tsao, JC, Lu, Q, Myers, CD, Kim, SC, Turk, N & Zeltzer, LK (2006b) Parent and child anxiety sensitivity: relationship to children's experimental pain responsivity. *Journal of Pain, 7*(5), 319-326. doi:10.1016/j.jpain.2005.12.004

Turner, JA, Whitney, C, Dworkin, SF, Massoth, D & Wilson, L (1995) Do changes in patient beliefs and coping strategies predict temporomandibular disorder

treatment outcomes? *Clinical Journal of Pain, 11*(3), 177-188. doi:10.1097/00002508-199509000-00004
Tutuncu, AC, Erbabacan, E, Dilmen Korkmaz, O, Ekici, B, Koksal, MG, Altintas, F & Kaya, G (2013) Effects of preemptive epidural infusion on cytokine response and postoperative pain in pediatric patients. [Pediatrik hastalarda preemptif epidural Infuzyonun postoperatif agri{dotless} ve sitokin cevaba etkisi.]. *Haseki Tip Bulteni, 51*(4), 162-167. doi:10.4274/Haseki.1015
van Tilburg, MAL, Claar, RL, Romano, JM, Langer, SL, Drossman, DA, Whitehead, WE, Abdullah, B & Levy, RL (2017) Psychological Factors May Play an Important Role in Pediatric Crohn's Disease Symptoms and Disability. *Journal of Pediatrics, 184*, 94-100.e101. doi:10.1016/j.jpeds.2017.01.058
VanDyck, R, Zitman, FG, Linssen, AC & Spinhoven, P (1991) Autogenic training and future oriented hypnotic imagery in the treatment of tension headache: outcome and process. *International Journal of Clinical & Experimental Hypnosis, 39*(1), 6-23. doi:10.1080/00207149108409615
Varni, JW, Nutakki, K & Swigonski, NL (2020) Cognitive functioning and pain interference mediate pain predictive effects on health-related quality of life in pediatric patients with Neurofibromatosis Type 1. *European Journal of Paediatric Neurology, 28*, 64-69.
Varni, JW, Shulman, RJ, Self, MM, Saeed, SA, Zacur, GM, Patel, AS, Nurko, S, Neigut, DA, Franciosi, JP, Saps, M, et al. (2018) Perceived medication adherence barriers mediating effects between gastrointestinal symptoms and health-related quality of life in pediatric inflammatory bowel disease. *Quality of Life Research, 27*(1), 195-204. doi:10.1007/s11136-017-1702-6
Venkatraghavan, L, Li, L, Bailey, T, Manninen, PH & Tymianski, M (2016) Sumatriptan improves postoperative quality of recovery and reduces postcraniotomy headache after cranial nerve decompression. *British Journal of Anaesthesia, 117*(1), 73-79. doi:10.1093/bja/aew152
VerLee, S, Butz, C, Ackerman, C, Burger, J, Fabia, R & Besner, G (2012, March) *The utility of virtual reality in minimizing procedural distress with pediatric burn patients.* Paper presented at the 44th Annual Meeting of the American Burn Association, Seattle, WA, United States.
Vo, T-T, Superchi, C, Boutron, I & Vansteelandt, S (2020) The conduct and reporting of mediation analysis in recently published randomized controlled trials: results from a methodological systematic review. *J Clin Epidemiol, 117*, 78-88. doi:10.1016/j.jclinepi.2019.10.001
Vos, T, Abajobir, AA, Abate, KH, Abbafati, C, Abbas, KM, Abd-Allah, F, Abdulkader, RS, Abdulle, AM, Abebo, TA, Abera, SF, et al. (2017) Global, regional, and national incidence, prevalence, and years lived with disability for 328 diseases and injuries for 195 countries, 1990–2016: a systematic analysis for the Global Burden of Disease Study 2016. *The Lancet, 390*(10100), 1211-1259. doi:https://doi.org/10.1016/S0140-6736(17)32154-2

Wall, BA (2000) *Social adjustment and perception of disease severity in children with juvenile rheumatoid arthritis.* ProQuest Dissertations Publishing. Retrieved from http://search.proquest.com/docview/304546925/

Wallace, D (2009) *Pain self-efficacy, coping behavior, somatic symptoms, and disability in young adults with headache: A path analysis.* ProQuest Dissertations Publishing. Retrieved from http://search.proquest.com/docview/304819284/

Walters, AS & Williamson, GM (1999) The role of activity restriction in the association between pain and depression: A study of pediatric patients with chronic pain. *Children's Health Care, 28*(1), 33-50. doi:10.1207/s15326888chc2801_3

Wand, BM, Abbaszadeh, S, Smith, AJ, Catley, MJ & Moseley, GL (2013) Acupuncture applied as a sensory discrimination training tool decreases movement-related pain in patients with chronic low back pain more than acupuncture alone: a randomised cross-over experiment. *British Journal of Sports Medicine, 47*(17), 1085-1089. doi:10.1136/bjsports-2013-092949

Wicksell, RK, Olsson, GL & Hayes, SC (2011) Mediators of change in acceptance and commitment therapy for pediatric chronic pain. *PAIN, 152*(12), 2792-2801.

Wong, RK, Yang, C, Song, GH, Wong, J & Ho, KY (2015) Melatonin regulation as a possible mechanism for probiotic (VSL#3) in irritable bowel syndrome: a randomized double-blinded placebo study. *Digestive Diseases & Sciences, 60*(1), 186-194. doi:10.1007/s10620-014-3299-8

Yang, M, Chen, X, Bo, L, Lao, L, Chen, J, Yu, S, Yu, Z, Tang, H, Yi, L, Wu, X, et al. (2017) Moxibustion for pain relief in patients with primary dysmenorrhea: A randomized controlled trial. *PLoS ONE, 12*(2), e0170952. doi:10.1371/journal.pone.0170952

Yang, W-Y, Liu, T-F, Chen, X-J, Guo, Y, Li, T, Qi, B-Q, Liu, F, Chang, L-X, Ruan, M & Liu, X-M (2020) Pharmacokinetics and pharmacodynamics of pegylated recombinant human granulocyte colony-stimulating factor in children with acute lymphoblastic leukemia: a prospective control trial. *Zhongguo Dang dai er ke za zhi= Chinese Journal of Contemporary Pediatrics, 22*(11), 1172-1177.

Zeidan, F, Adler-Neal, AL, Wells, RE, Stagnaro, E, May, LM, Eisenach, JC, McHaffie, JG & Coghill, RC (2016) Mindfulness-Meditation-Based Pain Relief Is Not Mediated by Endogenous Opioids. *Journal of Neuroscience, 36*(11), 3391-3397. doi:10.1523/JNEUROSCI.4328-15.2016

Ziadni, MS, You, DS, Johnson, L, Lumley, MA & Darnall, BD (2020) Emotions matter: The role of emotional approach coping in chronic pain. *European Journal of Pain, 24*(9), 1775-1784.

Zinman, LH, Sutton, D, Ng, E, Nwe, P, Ngo, M & Bril, V (2005) A pilot study to compare the use of the Excorim staphylococcal protein immunoadsorption system and IVIG in chronic inflammatory demyelinating polyneuropathy. *Transfusion and apheresis science, 33*(3), 317-324. doi:10.1016/j.transci.2005.07.010
